# Characterization of aging cancer-associated fibroblasts draws implications in prognosis and immunotherapy response in low-grade gliomas

**DOI:** 10.3389/fgene.2022.897083

**Published:** 2022-08-24

**Authors:** Zijian Zhou, Jinhong Wei, Lijun Kuang, Ke Zhang, Yini Liu, Zhongming He, Luo Li, Bin Lu

**Affiliations:** ^1^ Department of Neurosurgery, Qingdao Municipal Hospital, Qingdao University, Qingdao, China; ^2^ School of Basic Medical Sciences, Southwest Medical University, Luzhou, China; ^3^ Institutes for Systems Genetics, Frontiers Science Center for Disease-related Molecular Network, West China Hospital, Sichuan University, Sichuan, China

**Keywords:** low-grade glioma, aging cancer-associated fibroblasts, tumor microenvironment, prognosis, immunotherapy response

## Abstract

**Background:** Due to the highly variable prognosis of low-grade gliomas (LGGs), it is important to find robust biomarkers for predicting clinical outcomes. Aging cancer-associated fibroblasts (CAFs) within the senescent stroma of a tumor microenvironment (TME) have been recently reported to play a key role in tumor development. However, there are few studies focusing on this topic in gliomas.

**Methods and Results:** Based on the transcriptome data from TCGA and CGGA databases, we identified aging CAF-related genes (ACAFRGs) in LGGs by the weighted gene co-expression network analysis (WGCNA) method, followed by which LGG samples were classified into two aging CAF-related gene clusters with distinct prognosis and characteristics of the TME. Machine learning algorithms were used to screen out eight featured ACAFRGs to characterize two aging CAF-related gene clusters, and a nomogram model was constructed to predict the probability of gene cluster A for each LGG sample. Then, a powerful aging CAF scoring system was developed to predict the prognosis and response to immune checkpoint blockage therapy. Finally, the ACAFRGs were verified in two glioma-related external datasets. The performance of the aging CAF score in predicting the immunotherapy response was further validated in two independent cohorts. We also confirmed the expression of ACAFRGs at the protein level in glioma tissues through the Human Protein Atlas website and Western blotting analysis.

**Conclusion:** We developed a robust aging CAF scoring system to predict the prognosis and immunotherapy response in LGGs. Our findings may provide new targets for therapeutics and contribute to the exploration focusing on aging CAFs.

## Introduction

Low-grade gliomas (LGGs) encompassing grade II and III gliomas represent a group of primary tumors originating from the central nervous system and are very common in young adults compared to high-grade gliomas (grade IV, glioblastoma multiforme, GBM) (Perry and Wesseling, 2016). In 2016, the World Health Organization (WHO) updated the classification method for gliomas by combing histological diagnosis with molecular variations such as *IDH* mutation status and codeletion of the short arm of chromosome 1 and the long arm of chromosome 19 (1p/19q codeletion) (Komori, 2017). Previous studies revealed that glioma patients with mutant *IDH* exhibited a more favorable response to current therapy including radiation and chemotherapy, implying the correlation between molecular alterations and prognosis (Cairncross et al., 2014). Due to high heterogeneity, glioma patients had various clinical outcomes even with the same diagnosis. While LGG patients tend to get a longer survival time with the median overall survival ranging from 5.6–13.3 years, the prognosis of LGG patients can be highly variable (Hottinger et al., 2016; Ostrom et al., 2018). Exploration focusing on biomarkers for predicting prognosis is becoming a hot spot in cancer research.

Most of the previous investigations have been focusing on tumor cells themselves while pioneering studies have claimed the significant importance of the crosstalk between tumor cells and the surrounding microenvironment in the course of tumor development (Radin and Tsirka, 2020). As a complex environment with dynamic alterations, the tumor microenvironment (TME) represents the non-tumoral components around tumor cells, including the extracellular matrix (ECM) and various cell populations such as immune cells, fibroblasts, and endothelial cells (Anderson and Simon, 2020). Fibroblasts, which constitute a major proportion of the TME, refer to a heterogeneous cell population derived from mesenchymal lineage cells and are collectively defined as cancer-associated fibroblasts (CAFs) (Piersma et al., 2020b). The past few years have witnessed significant strikes in the explorations of CAFs. Considering its well-established roles in epithelial–mesenchymal transition (EMT) (Erin et al., 2020) and maintenance of cancer stemness (Su et al., 2018), which is important for tumorigenesis and progression, CAFs are known to be closely related to prognosis in a variety of cancers (Miyai et al., 2020). Recently, T-cell-targeted immunotherapy is emerging as a robust treatment option for intractable cancers. As a novel type of immunotherapy, immune checkpoint blockage treatment such as CTLA4 and PD-1/PD-L1 antibodies has demonstrated pronounced success by activating T cells (Topalian et al., 2015). However, only a minority of patients get a favorable response to immunotherapy (Pitt et al., 2016). The T cell’s capacity to kill tumor cells is significantly affected by the tumor stromal microenvironment, in which CAFs are understood to be a key player in immunosuppressive activity and reduce the efficacy of immune checkpoint blockage treatment (Baker et al., 2021; Miyai et al., 2022). Over the past decade, cancer has been usually recognized as a disease of aging and CAFs appear to be easily influenced by this age-related effect (Fane and Weeraratna, 2020). Despite the fact that senescence can be caused by tumor-independent manners, the senescence of CAFs is generally induced by signaling from tumor cells (Sahai et al., 2020). Senescent CAFs in the TME and their secretory profile (senescence-associated secretory phenotype, SASP) are known to influence all aspects of tumor development, including tumor initiation, progression, anti-tumor immunity, and chemoresistance (Yasuda et al., 2021b; Ruhland and Alspach, 2021). Overall, comprehensive analysis of aging CAFs is meaningful to determine biomarkers for predicting prognosis and immunotherapy response in gliomas. However, the explanation for the complicated roles of aging CAFs in the TME is hindered due to the lack of specific biomarkers to identify both the aging status and the cell type of CAFs *in vivo (* *Sahai et al., 2020* *)*. Moreover, there are no studies focusing on aging CAF-related genes or prediction models in gliomas at this time.

In this research, we identified aging CAF-related genes (ACAFRGs) by comprehensive analysis of the transcriptome data from TCGA and CGGA databases, based on which two distinct aging CAF-related gene clusters were determined. Subsequently, we constructed an aging CAF scoring system to predict the prognosis and immunotherapy response for LGG patients. Finally, we confirmed the expression of the aging CAF-related genes at the protein level. Our study may shed light on the exploration of aging CAFs and contribute to the development of aging CAF-targeted therapy for glioma patients in the future.

## Materials and methods

### Data acquisition

A dataset containing 508 LGG samples with the corresponding RNA sequencing (RNA-seq) data was downloaded from TCGA database (The Cancer Genome Atlas, http://cancergenome.nih.gov/). The annotation file, Genome Reference Consortium Human Build 38 (GRCh38), which was acquired from the Ensembl website (http://asia.ensembl.org/), was employed to annotate the RNA-seq data. The transcriptome data (dataset ID: mRNA-array_301) composed of 159 LGG samples were obtained from the CGGA database (Chinese Glioma Genome Atlas, http://cgga.org.cn/index.jsp) (Fang et al., 2017; Wang et al., 2017). The corresponding clinical information for LGG patients involved in the two datasets was also downloaded from the aforementioned websites. R software (version 4.1.1) was utilized for the bioinformatic analysis and visualization of the data.

### Determination of ACAFRGs

In our study, the transcriptome data from TCGA database were first transformed to transcripts per million (TPM) values for further combination with the transcriptome data from the CGGA database. Robust multi-array average normalization would be performed for the transcriptome data by the normalizeBetweenArrays function in the limma R package (Smyth et al., 2005) when the distribution of gene expression values in the transcriptome data from different databases was not uniform, followed which quantile normalization and log2 transformation were carried out. Combat function in the sva R package (Leek et al., 2012) was used to remove the batch effect caused by non-biotechnological bias when merging the transcriptome data. In addition, two-dimensional principal component analysis (PCA) cluster plots were utilized to show the sample distribution before and after batch effect correction.

The stromal score which indicated the stromal components of the TME for each LGG sample was calculated by the ESTIMATE algorithm (Estimation of STromal and Immune cells in MAlignant Tumor tissues using Expression data) through the estimate R package (Yoshihara et al., 2013) based on the gene expression values in the merged transcriptome data. Furthermore, LGG samples in the merged data were separated into high- and low-stromal score groups according to the optimal cut-off value which was determined through survminer and survival R packages. The differentially expressed genes (DEGs) between the high- and low-stromal score groups were screened out by |log_2_ FC (fold change)| > 0.5 and adjusted *p*-values (FDR, false discovery rate) < 0.05 through the limma R package. The robust DEGs were considered as stromal cell-related genes. The relative abundance of fibroblasts in the TME was quantified *via* the MCP counter (Becht et al., 2016). Based on the expression profiles of stromal cell-related genes, weighted gene co-expression network analysis (WGCNA) (Zhang and Horvath, 2005) was utilized to determine the ACAFRGs by using the WGCNA R package. To construct the network, we first calculated the robust correlations between all the stromal cell-related genes across all LGG samples in the data. The optimal power parameter was set to amplify the strong connections between genes in the same gene modules and to penalize the weak connections between genes in different modules. In this study, the optimal power value was determined when the scale independence *R* ^2^ was higher than 0.90 and the mean connectivity degree of the co-expression network was relatively higher. A total of four phenotypes, namely, survival time, age, fibroblasts, and stromal score were involved in WGCNA. The Spearman method was used to analyze the correlation between MEs and phenotypes.

### Aging CAF-related gene clusters

First, ACAFRGs with prognostic values were screened out *via* univariate Cox regression analysis by using the survival R package, in which *p* < 0.05 was considered statistically significant. Then, distinct aging CAF-related gene clusters were determined by a consensus clustering method using the ConsensusClusterPlus R package based on the expression profiles of prognostic ACAFRGs in the merged data (Wilkerson and Hayes, 2010). Our clustering analysis was based on the Partitioning Around Medoid (PAM) algorithm which was derived from the k-means machine learning algorithm. A total of 50 repetitions were conducted in the consensus clustering process for the stability of our classification and 80% of the LGG samples were involved in each iteration. The optimal number for the subgroup assignment was determined based on the consensus matrix heatmap and the relative change values of the area under the cumulative distribution function (CDF) curves.

### Identification of the featured ACAFRGs for discriminating aging CAF-related gene clusters

Based on the expression profiles of ACAFRGs, two machine learning algorithms were adopted to select the key genes for discriminating aging CAF-related gene clusters, namely, least absolute shrinkage and selection operator (LASSO) logistic regression (Tibshirani, 1996) and support vector machine-recursive feature elimination (SVM-RFE) (Suykens and Vandewalle, 1999). The LASSO algorithm serves as a special instance of the penalized least squares regression with the L1-penalty function. LASSO logistic regression was carried out by using the glmnet R package, in which the optimal number of featured genes was determined when the lambda value was minimal. The SVM-RFE machine learning algorithm was performed with five-fold cross-validation by using the e1071 R package, in which the optimal number of featured genes was determined when the root mean square error (RMSE, cross-validation) was minimal. Afterward, the overlapping featured genes were selected for further analysis. Furthermore, the random forest (RF) machine learning algorithm was used to further screen out featured genes *via* the randomForest R package (Breiman, 2001), in which ntrees and mtry were set at 500 and 3, respectively. First, the optimal number of the random forest trees was determined when the cross-validation error presented minimal. Then, the random forest with the optimal number of trees was constructed. To obtain featured genes with high importance, the importance of each gene was calculated. The genes with importance >10 were selected as the featured genes for aging CAF-related gene clusters.

Based on the expression profiles of the featured ACAFRGs, we constructed a nomogram model to predict aging CAF-related gene cluster A. Calibration curves, decision curve analysis (DCA), and clinical impact curve were used to evaluate the performance of the model to predict aging CAF-related gene cluster A.

### Aging CAF scoring system

Univariate cox regression analysis was implemented to determine whether the ACAFRGs were positively or negatively associated with prognosis, according to which the ACAFRGs were divided into favorable and unfavorable genes. Gene set variation analysis (GSVA) can quantify the enrichment with respect to specific functions or characteristics for individuals based on specific gene sets and transcriptome data (Hänzelmann et al., 2013). GSVA and single-sample gene set enrichment analysis (ssGSEA) were used to produce GSVA scores regarding the unfavorable and favorable gene sets for LGG samples by using the GSVA R package (Hänzelmann et al., 2013). First, the gene expression values in the transcriptome data were sequenced to obtain their rank. Then, the genes in the unfavorable and favorable gene sets were extracted, followed by which the expression levels of unfavorable and favorable genes were summed. Finally, we obtained the enrichment scores of unfavorable and favorable gene sets for each LGG sample. The aging CAF score for each LGG sample was calculated by the following formula: agingCAFscore=GSVAscoreA−GSVAscoreB , in which GSVAscoreA represents the enrichment regarding unfavorable ACAFRGs and GSVAscoreB represents the enrichment regarding favorable ACAFRGs. Subsequently, LGG samples were classified into high- and low-aging CAF score groups according to the optimal cut-off value of aging CAF scores which was determined by survminer and survival R packages. Moreover, the aging CAF score and other common clinicopathological characteristics were taken into consideration for the construction of a nomogram model to predict the prognosis of LGG patients by using the rms R package.

### Differential function enrichment analysis

Kyoto Encyclopedia of Genes and Genomes (KEGG) pathways and molecular functions (GO, Gene Ontology) between subgroups were analyzed by using GSVA, GSEABase, and limma R packages, in which the reference gene sets including “c5.go.mf.v7.4.symbols.gmt” and “c2.cp.kegg.v7.4.symbols.gmt” were downloaded from the GSEA database. First, the GSVA score for each function term was calculated for each sample based on the reference gene sets and the gene expression profiles. The function terms with |log_2_ FC| > 0.1 and adjusted *p*-values (FDR) < 0.05 between the two groups were then screened out and were considered differentially enriched. In the heatmaps, the values of the GSVA score were centered and scaled in the row direction, in which the rows are scaled to have mean zero and standard deviation one (z score). The top 20 differentially enriched function terms were shown in the heatmaps.

### Exploration of the TME

The immune and stromal components of the TME were quantified *via* the ESTIMATE algorithm (Yoshihara et al., 2013). The relative abundance of essential immune and stromal cells in the TME was quantified *via* the MCP counter (Becht et al., 2016). CIBERSORT, a deconvolution algorithm based on linear support vector regression, was employed to further calculate the abundance of infiltrating immune cells in the TME based on the gene expression profiles of LGG samples (Newman et al., 2015). The SsGSEA method was also employed to quantify the immune cells based on the input immune cell-related gene set. In addition, we downloaded the results of the abundance of critical cells in the TME for all LGG samples from TCGA on the TIMER2.0 website (http://timer.cistrome.org/), including XCELL, TIMER, QUANTISEQ, MCP counter, EPIC, CIBERSORT, and CIBERSORT ABS. Tumor Immune Dysfunction and Exclusion (TIDE)-related scores for LGG samples were calculated to explore the immunotherapeutic response (http://tide.dfci.harvard.edu/).

### Genetic mutation analysis

The genetic mutation data for LGG samples were retrieved from TCGA database. The maftools R package was used for the analysis of somatic variants. The cumulative nonsynonymous mutations per million bases in coding regions were defined as tumor mutation burdens (TMBs). LGG samples with exome nonsynonymous mutations were taken into account.

### Validation in external datasets

Two independent datasets from the CGGA database (dataset ID: mRNAseq_325 and mRNAseq_693) were chosen as validation cohorts (Bao et al., 2014; Wang et al., 2015; Zhao et al., 2017; Liu et al., 2018; Zhao et al., 2021) to verify the aging CAF-related gene cluster and aging CAF score. In addition, the aging CAF score was further validated in two external datasets (GSE78220 and IMvigor210 cohort) to explore the performance in predicting the immunotherapeutic response (Hugo et al., 2016; Balar et al., 2017; Mariathasan et al., 2018).

### Validation of the ACAFRGs at the protein level

A total of four ACAFRGs, namely, RBP1, PDPN, FKBP9, and MSN were randomly selected from the featured ACAFRGs. The differential expression patterns of the aforementioned genes between normal and glioma tissues were explored on the Human Protein Atlas website (https://www.proteinatlas.org/).

Western blotting was implemented to further verify the differential expression levels of the featured ACAFRGs between normal and glioma tissues. Normal brain tissues were acquired from patients with epilepsy who received temporal lobe resection. Glioma tissues which were histologically diagnosed as grades II (G2) and III (G3) were obtained from patients who received tumor resection. In this study, three normal samples, six G2 glioma samples, and nine G3 glioma samples were involved. One normal sample, two G2 glioma samples, and three G3 glioma samples were taken together for Western blotting analysis every time. The optical density of the bands in Western blotting was analyzed by using ImageJ software (Software Version: 1.53q, Wayne Rasband and contributors, National Institutes of Health, United States). The differential analysis between different samples was conducted by the limma R package.

The collected tissues were separately homogenized and lysed in RIPA lysis buffer containing protease and phosphatase inhibitors at 0–4°C. The homogenized protein samples were centrifuged at 1,000 g for 15 min at 4°C to obtain the protein in the cytoplasm. A Bio-Rad protein assay kit was used to correct the protein content to an equal level. The protein samples were homogenized with the prepared loading buffer and then boiled for 5 min at 100°C. The same amounts of protein samples were added to SDS-PAGE and electrophoresed at 80 V for 1 h. Afterward, the protein was transferred to polyvinylidene difluoride (PVDF) membranes at 50 V for 1 h. The primary antibodies used in this study were as follows: RBP1, Podoplanin (PDPN), FKBP9, TIMP1, CHI3L1, moesin (MSN), and *β*-actin. After incubation with the primary antibodies for 12 h, the membranes were then incubated with the secondary anti-rabbit or anti-mouse horseradish peroxidase (HRP) antibodies. Finally, the membranes were visualized by enhanced chemiluminescence (ECL) solution.

### Statistical analysis

The prognosis between different subgroups was compared using Kaplan–Meier survival analysis by survminer and survival R packages, in which the log-rank test was utilized for statistical analysis. Comparisons between the two groups were carried out by using Wilcoxon rank-sum tests. Comparisons of categorical variables between two groups were presented by chi-square tests. Comparisons of continuous variables between two groups were conducted *via* an independent Student’s t-test. Two-tailed *p* < 0.05 was considered statistically significant.

## Results

### Determination of ACAFRGs

**FIGURE 1:**
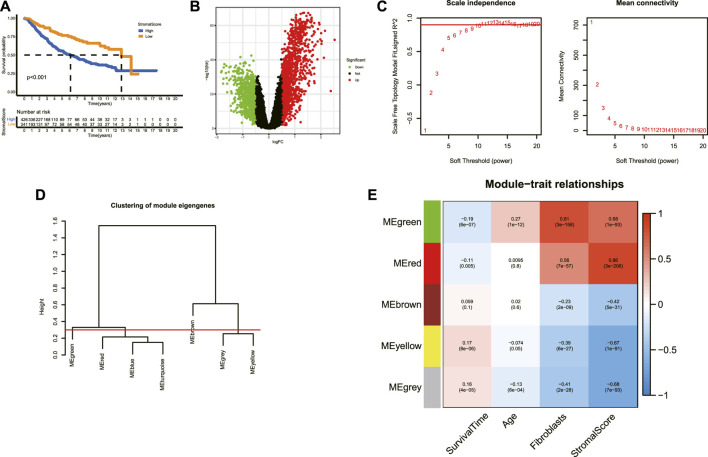
Determination of ACAFRGs. **(A)** Kaplan–Meier survival analysis for LGG patients assigned to high and low stromal scores (*p* < 0.001). **(B)** Volcano plot of the DEGs between high- and low-stromal score groups. Genes with |log_2_ FC (fold change)| > 0.5 and adjusted *p*-values (FDR, false discovery rate) < 0.05 were considered significant. Green dots represent downregulated genes in the high-stromal score group and red dots represent upregulated genes in the high-stromal score group. **(C)** Scale independence index and mean connectivity values when soft threshold (power) ranges from 1–20. The red line was set at 0.90. **(D)** Clustering of the module eigengenes. The cut height was set at 0.30 as depicted with the red line. **(E)** Heatmap demonstrating the key gene modules associated with survival time, age, fibroblasts, and stromal scores. Pearson correlation coefficients and *p*-values were shown in cells. ACAFRGs, aging cancer-associated fibroblast related genes; PCA, principal component analysis; LGG, low-grade glioma; DEGs, differentially expressed genes.

The schematic diagram of the workflow of this study is shown in Supplementary Figure S1. The corresponding clinicopathological information for LGG samples in the merged data is demonstrated in Supplementary Tables S1, S2. The inter-batch difference was corrected when merging the transcriptome data from TCGA and CGGA databases. As illustrated in the two-dimensional PCA cluster diagram (Supplementary Figures S2A,B), the inter-batch difference was removed. The Kaplan–Meier survival analysis indicated that the high-stromal score group tended to get a worse prognosis compared to the low score group (Figure 1A). As shown in the volcano plot (Figure 1B), the robust DEGs between the high and low-stromal score groups were screened out for further analysis. Given that CAFs represent a major component of the TME and accumulate in the tumor stroma across multiple cancers (Kalluri, 2016; Kobayashi et al., 2019; Piersma et al., 2020a; Miyai et al., 2020), the stroma-related DEGs were selected for the identification of CAF-related genes. Based on the expression profiles of stroma-related genes, a co-expression network was constructed by the WGCNA method. As shown in Figure 1C, the scale independence *R* ^2^ increased and the mean connection decreased when the soft threshold (power) value increased. The optimal power parameter was set at 10 to amplify the strong connections between genes in the same gene modules and to penalize the weak connections between different modules (scale independence *R* ^2^ = 0.89, mean connectivity = 7.02). The genes within the same module presented significant within-module connectivity and were defined as the hub genes for the specific modules (Figure 1D, Supplementary Figure S2C). The correlation between module eigengenes (MEs) and phenotypes including survival time, age, fibroblasts, and stromal score were subsequently analyzed (Figure 1E). A total of five gene modules were identified, in which the green module was positively associated with age (*r* = 0.27, p = 1e-12) and fibroblasts (*r* = 0.81, p = 3e-158) and the gray module was negatively associated with age (*r* = −0.13, p = 6e-04) and fibroblasts (*r* = −0.41, p = 2e-28). A total of 463 genes in the two modules were determined as ACAFRGs (Supplementary Table S3).

### Aging CAF-related gene clusters

**FIGURE 2:**
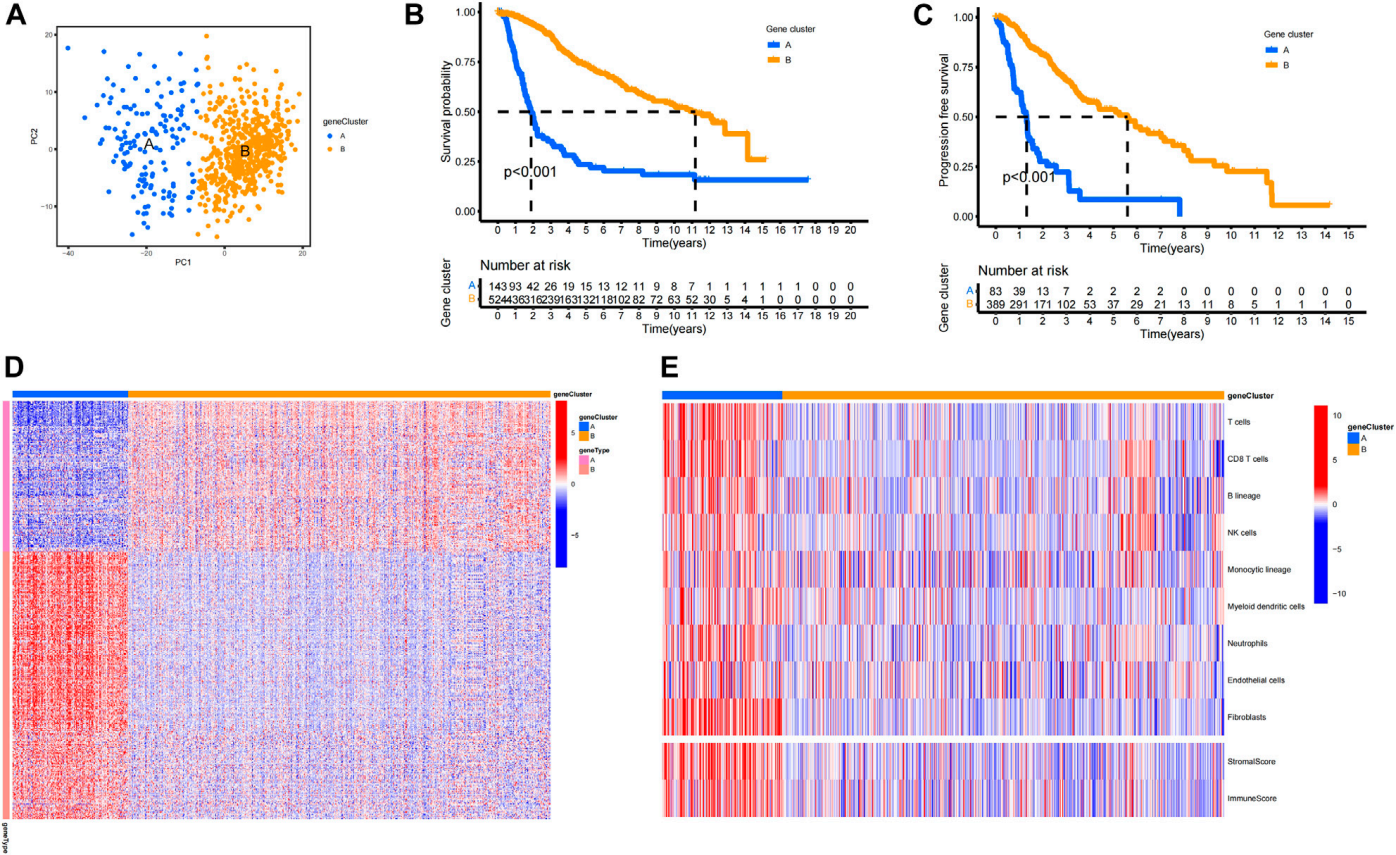
Aging CAF-related gene clusters. **(A)** PCA of LGG samples based on the expression profiles of ACAFRGs. Blue dots represent samples of gene cluster A and yellow dots represent samples of gene cluster B. **(B)** Kaplan–Meier analysis indicated that gene cluster A had a shorter overall survival than gene cluster B (*p* < 0.001). **(C)** Kaplan–Meier analysis indicated that gene cluster A had shorter progression-free survival than gene cluster B (*p* < 0.001). **(D)** Heatmap displaying the distinct expression patterns of ACAFRGs between two gene clusters, in which gene type A was upregulated in gene cluster B while gene type B was upregulated in gene cluster A. **(E)** Heatmap displaying the distinct characteristics of the TME between two gene clusters. ACAFRGs, aging cancer-associated fibroblast related genes; LGG, low-grade glioma; PCA, principal component analysis.

A total of 400 ACAFRGs with prognostic values were screened out *via* univariate Cox regression analysis. LGG samples in the merge data were classified into two gene clusters based on the expression profiles of prognostic ACAFRGs (Supplementary Figure S3A). As shown in the heatmap of the consensus matrix, samples were reasonably classified into two gene clusters (*k* = 2), in which samples with high consensus scores between them were more likely to be grouped into the same cluster. Moreover, no apparent increase was found in the area under the CDF curve when *k* = 2 (relative change <0.4). PCA confirmed the results of the subgroup assignment (Figure 2A). Kaplan–Meier analysis revealed that gene cluster A had shorter overall survival and shorter progression-free survival than gene cluster B, indicating that LGG samples in gene cluster A tended to get a worse prognosis (Figures 2B,C). The prognostic ACAFRGs were separated into gene types A and B, in which ACAFRGs in gene type A were downregulated in gene cluster A and upregulated in gene cluster B while ACAFRGs in gene type B were upregulated in gene cluster A and downregulated in gene cluster B (Figure 2D). In addition, the stromal and immune scores of gene cluster A were significantly higher than those of gene cluster B (Supplementary Figures S3B,C). The abundance of most types of the cells in the TME was higher in gene cluster A, including T cells, CD8 T cells, epithelial cells, fibroblasts, and macrophages (Figure 2E, Supplementary Figures S3B,C).

### Identification of the featured ACAFRGs for discriminating aging CAF-related gene clusters

**FIGURE 3:**
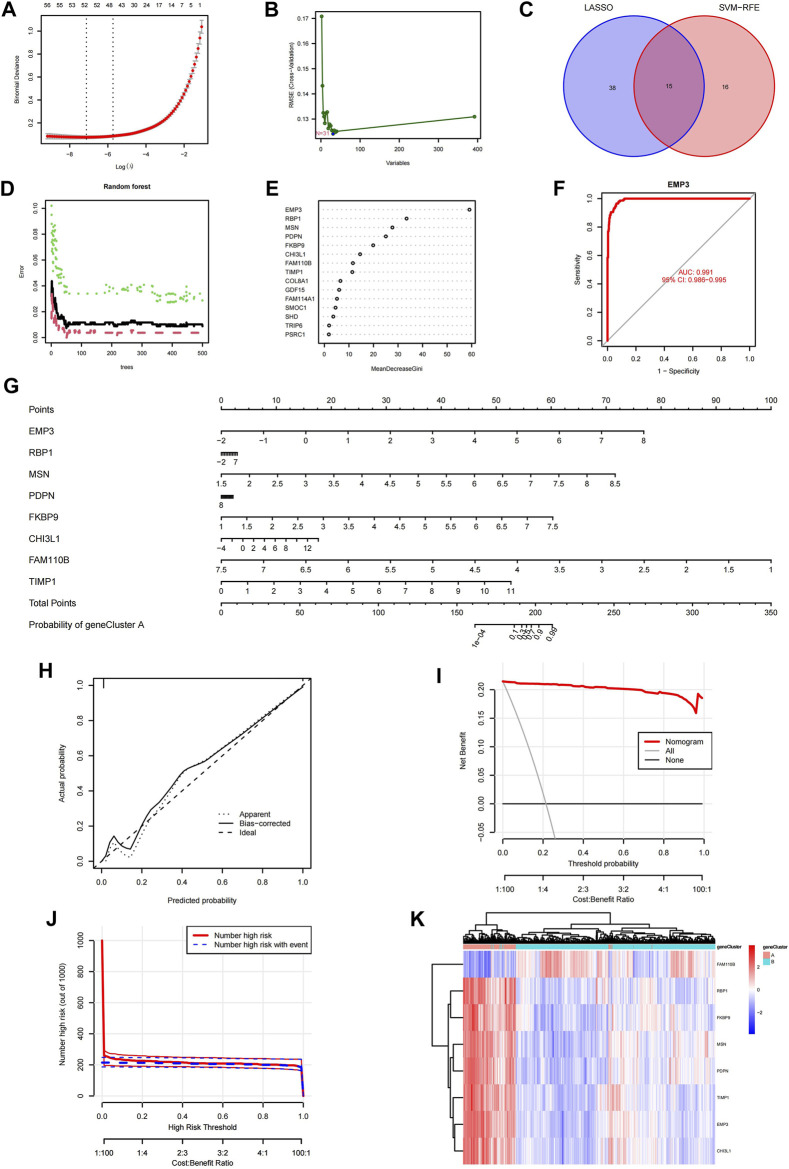
Identification of the featured ACAFRGs for discriminating aging CAF-related gene clusters. **(A)** Determination of the optimal number of featured ACAFRGs by LASSO logistic regression, in which 53 featured ACAFRGs were determined when the lambda value was minimal. **(B)** Determination of the optimal number of featured ACAFRGs by using the SVM-RFE algorithm (*N* = 31). **(C)** Venn plot showing the 15 overlapped featured ACAFRGs obtained by the aforementioned methods. **(D)** Optimal number of the random forest trees was determined when the cross-validation error presented minimal. The red dots represent the samples in gene cluster A, the green dots represent the samples in gene cluster B, and the black dots represent all the samples. **(E)** Importance of the featured genes in which eight ACAFRGs with importance higher than 10 were selected as the optimal featured ACAFRGs. **(F)** ROC curve demonstrating the accuracy of the featured genes for discriminating two gene clusters (take EMP3 for example, AUC value = 0.991). **(G)** Construction of the nomogram model based on the eight featured ACAFRGs to calculate the probability of gene cluster A for each sample. **(H)** Calibration curve revealed the accuracy of the nomogram model. **(I)** DCA of the nomogram model. **(J)** Clinical impact curves of the nomogram model. **(K)** Unsupervised clustering of samples based on the expression of the eight featured ACAFRGs. ACAFRGs, aging cancer-associated fibroblast related genes; CAF, cancer-associated fibroblast; LASSO, least absolute shrinkage and selection operator; SVM-RFE, support vector machine-recursive feature elimination; ROC, receiver operating characteristic; AUC, area under curve; DCA, decision curve analysis.

First, the LASSO logistic regression machine learning method was utilized to identify the featured ACAFRGs for discriminating two aging CAF-related gene clusters, in which 53 featured ACAFRGs were determined when the lambda value was minimal (Figure 3A). Subsequently, the SVM-RFE machine learning algorithm was performed to further determine the featured ACAFRGs, in which 31 featured ACAFRGs were identified when RMSE was minimal (Figure 3B). We obtained 15 overlapped genes *via* the two aforementioned methods (Figure 3C). Moreover, the random forest model was used to further screen out featured ACAFRGs based on the expression profiles of the aforementioned 15 featured ACAFRGs, in which 50 trees were determined when the cross-validation error presented minimal (Figure 3D). Based on the determination of the optimal number of forest trees, the importance of each gene was calculated, followed by which eight ACAFRGs with importance higher than 10 were selected as the optimal featured ACAFRGs for discriminating aging CAF-related gene clusters (Figure 3E). ROC curves revealed the efficacy of each featured gene for discriminating aging CAF-related gene clusters (Figure 3F, Supplementary Figure S4), in which all the AUC values were higher than 0.930. A nomogram model combing the eight featured ACAFRGs was constructed to predict aging CAF-related gene cluster A (Figure 3G). The calibration curves indicated a good performance of the nomogram model to predict gene cluster A (Figure 3H). The red line in the DCA remained above the gray and black lines from 0 to 1, suggesting that the decisions based on the nomogram model were accurate (Figure 3I). The clinical impact curve confirmed the robust performance of the nomogram model (Figure 3J). Unsupervised clustering for LGG samples was conducted based on the expression of the eight featured ACAFRGs. We found that samples in the same gene cluster tended to be aggregated together, indicating that LGG samples could be well distinguished through the expression of the eight featured ACAFRGs (Figure 3K). Additionally, the differential expression patterns of the eight featured genes between gliomas and normal samples were analyzed through the GEPIA online tools (Li et al., 2021) (GEPIA, Gene Expression Profiling Interactive Analysis, http://gepia.cancer-pku.cn/). As shown in Supplementary Figure S5, most of the featured ACAFRGs were upregulated in gliomas compared to normal brain samples, except for FAM110B with no significant difference.

### Comparison of the prognosis between low- and high-aging CAF score groups

**FIGURE 4:**
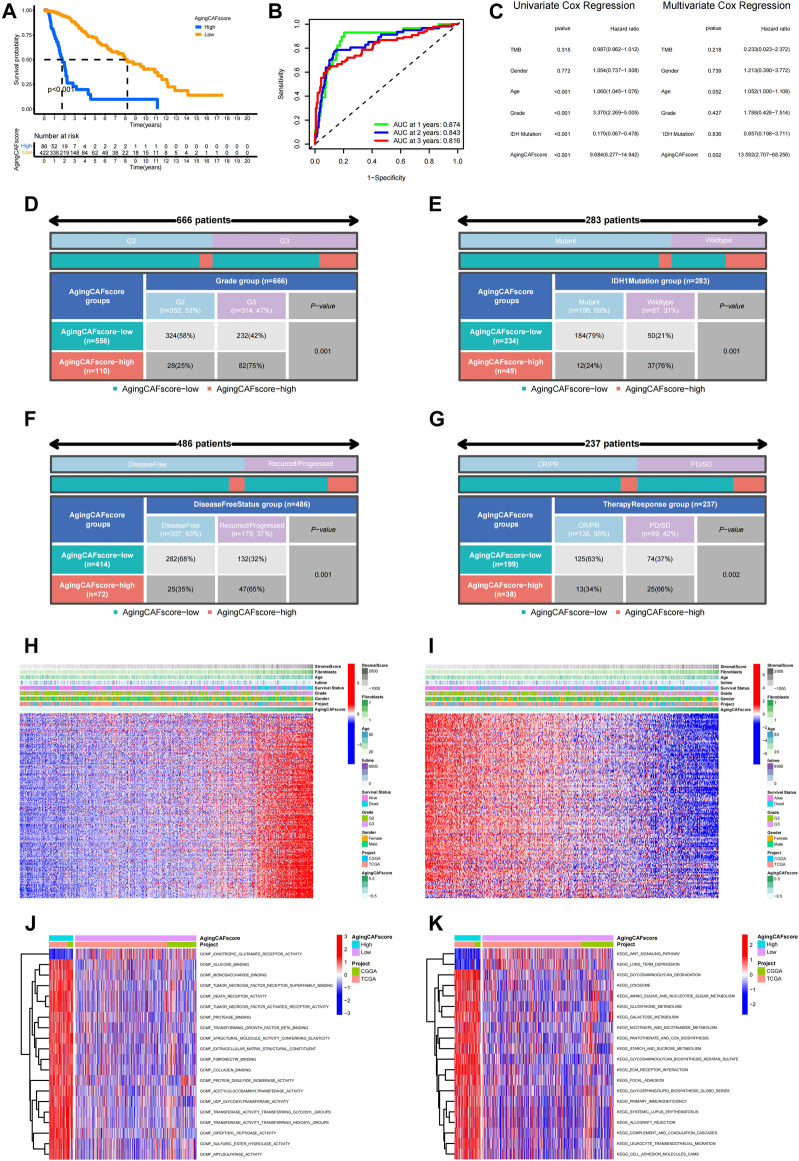
Comparison of the prognosis between low- and high-aging CAF score groups. **(A)** Kaplan–Meier survival analysis demonstrated that the high-aging CAF score group had a worse prognosis than the low-aging CAF score group in TCGA cohort (*p* < 0.001). **(B)** Time-dependent ROC curves of the aging CAF score in TCGA cohort. **(C)** Univariate/multivariate Cox regression analysis of the aging CAF score in TCGA cohort. **(D–G)** Correlation analysis between the aging CAF score group and grade **(D)**, IDH1 mutation status **(E)**, disease-free status **(F)**, and conventional therapy response **(G)**. All *p*-values less than 0.05. **(H)** Heatmap showing the expression patterns of unfavorable ACAFRGs with increasing aging CAF scores. **(I)** Heatmap showing the expression patterns of favorable ACAFRGs with increasing aging CAF scores. **(J)** Differentially enriched molecular functions between low- and high-aging CAF score groups. **(K)** Differentially enriched KEGG pathways between low- and high-aging CAF score groups. The function terms with |log_2_ FC| > 0.1 and adjusted *p*-values (FDR) < 0.05 between two groups were considered differentially enriched. The values of the GSVA score for function terms were centered and scaled in the row direction. The top 20 differentially enriched function terms were shown in the heatmaps. CAF, cancer-associated fibroblast; ROC, receiver operating characteristic; AUC, area under curve; ACAFRGs, aging cancer-associated fibroblast-related genes; CR/PR, complete remission/partial remission; PD/SD, progressed disease/stable disease, GSVA, gene set variation analysis.

Based on the expression profiles of the prognostic ACAFRGs, the aging CAF score was calculated for each LGG sample in the merged data through the GSVA method. LGG samples were then divided into low- and high-aging CAF score groups. Kaplan–Meier survival analysis suggested that the high-aging CAF score group exhibited a worse prognosis than the low-score group in TCGA cohort (Figure 4A). ROC curves revealed that the accuracy of the aging CAF score was 0.874, 0.843, and 0.816 when predicting the 1, 2, and 3-year overall survival of LGG samples, respectively, in TCGA cohort (Figure 4B). Univariate Cox regression analysis indicated that the aging CAF score was significantly correlated with the prognosis of LGG samples and multivariate Cox regression analysis demonstrated that the aging CAF score served as an independent prognostic factor in TCGA cohort (Figure 4C). Similar results were acquired in the CGGA cohort (Supplementary Figures S6A–C). The relationship between the aging CAF score and multiple clinicopathological characteristics was explored in our research. We found that the proportion of glioma patients of grade III (G3) was significantly higher in the high-aging CAF score group than those in the low-score group (Figure 4D). IDH1 mutation was more frequent in the low-aging CAF score group (Figure 4E). Gliomas were more likely to recur or progress in the high-aging CAF score group than in the low-score group (Figure 4F). With respect to the therapeutic response to conventional treatment, more patients got complete or partial remission in the low-aging CAF score group compared to the high-score group (Figure 4G). Based on clinical features such as age, gender, and grade, LGG samples were stratified into different subgroups. We compared the aging CAF scores between subgroups with different clinical features. The aging CAF scores of old individuals were significantly higher than those of young individuals and the scores of samples with G3 were higher compared to G2. There was no correlation between the aging CAF score and gender (Supplementary Figure S7A). The overall survival of the high-aging CAF score group was substantially shorter than those of the low-score group even though samples were separated into subgroups with different clinical features (Supplementary Figure S7B). All these findings suggested that patients in the high-aging CAF score group tended to get a poor prognosis.

Consistent with the aforementioned results, we found that most samples of gene cluster A belonged to the high-aging CAF score group and all samples of gene cluster B were classified into the low-aging CAF score group (Supplementary Figure S6D). In addition, the aging CAF scores of gene cluster A were significantly higher than those of gene cluster B (Supplementary Figure S6E).

We further probed into the correlation between the expression patterns of ACAFRGs and aging CAF scores. As shown in Figures 4H,I, the expression levels of unfavorable ACAFRGs were upregulated with the increase in the aging CAF score while the expression levels of favorable ACAFRGs were downregulated with the increase in the aging CAF score. Differentially enriched functions between the two aging CAF score groups were analyzed to explore the underlying molecular mechanisms. We found that stroma-related functions were active in the high-aging CAF score group, including fibronectin binding, collagen binding, ECM receptor interaction, focal adhesion, and cell adhesion molecule (CAM)-related pathways, which may contribute to tumorigenesis and progression (Walker et al., 2018; Winkler et al., 2020; Bhargav et al., 2022) (Figures 4J,K).

### Construction of a nomogram model

**FIGURE 5:**
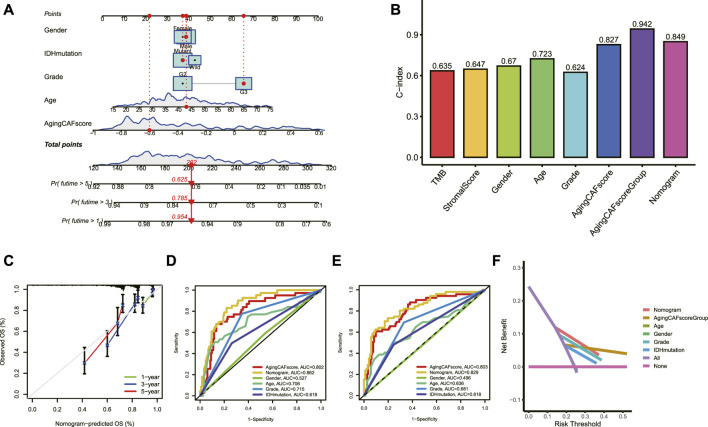
Construction of a nomogram model. **(A)** Nomogram model based on the aging CAF score and multiple clinicopathological factors was constructed. **(B)** Values of the C-index for the nomogram model and aging CAF score group were higher than those of other clinical factors. **(C)** Calibration curves for the nomogram model. **(D,E)** ROC curves of the nomogram model for predicting the 2 **(D)** and 3-year **(E)** overall survival. **(F)** DCA of the nomogram model for predicting the prognosis at 3 years. CAF, cancer-associated fibroblast; C-index, consistency index; ROC, receiver operating characteristic; AUC, area under curve; DCA, decision curve analysis.

A nomogram model based on the aging CAF score and multiple clinicopathological factors was constructed to improve the predictive ability for prognosis (Figure 5A). As shown in Figure 5B, the values of the C-index for the aging CAF score, aging CAF score group, and nomogram model were 0.827, 0.942, and 0.849, respectively, indicating a good performance (the C-index of 0.5 represents a random chance and 1.0 represents ideal ability to predict the prognosis). The calibration curves of the nomogram model presented a good agreement between the prediction and the actual observation (Figure 5C). The AUC values of ROC curves for the nomogram model were 0.862 and 0.829 when predicting the 2 and 3-year overall survival, respectively, indicating the high accuracy of the model (Figures 5D,E). The results of DCA demonstrated that the nomogram model got great net benefits across a large range of risk thresholds (Figure 5F). All these findings revealed the powerful performance of the nomogram model.

### Exploration of the correlation between the aging CAF score and TME

**FIGURE 6:**
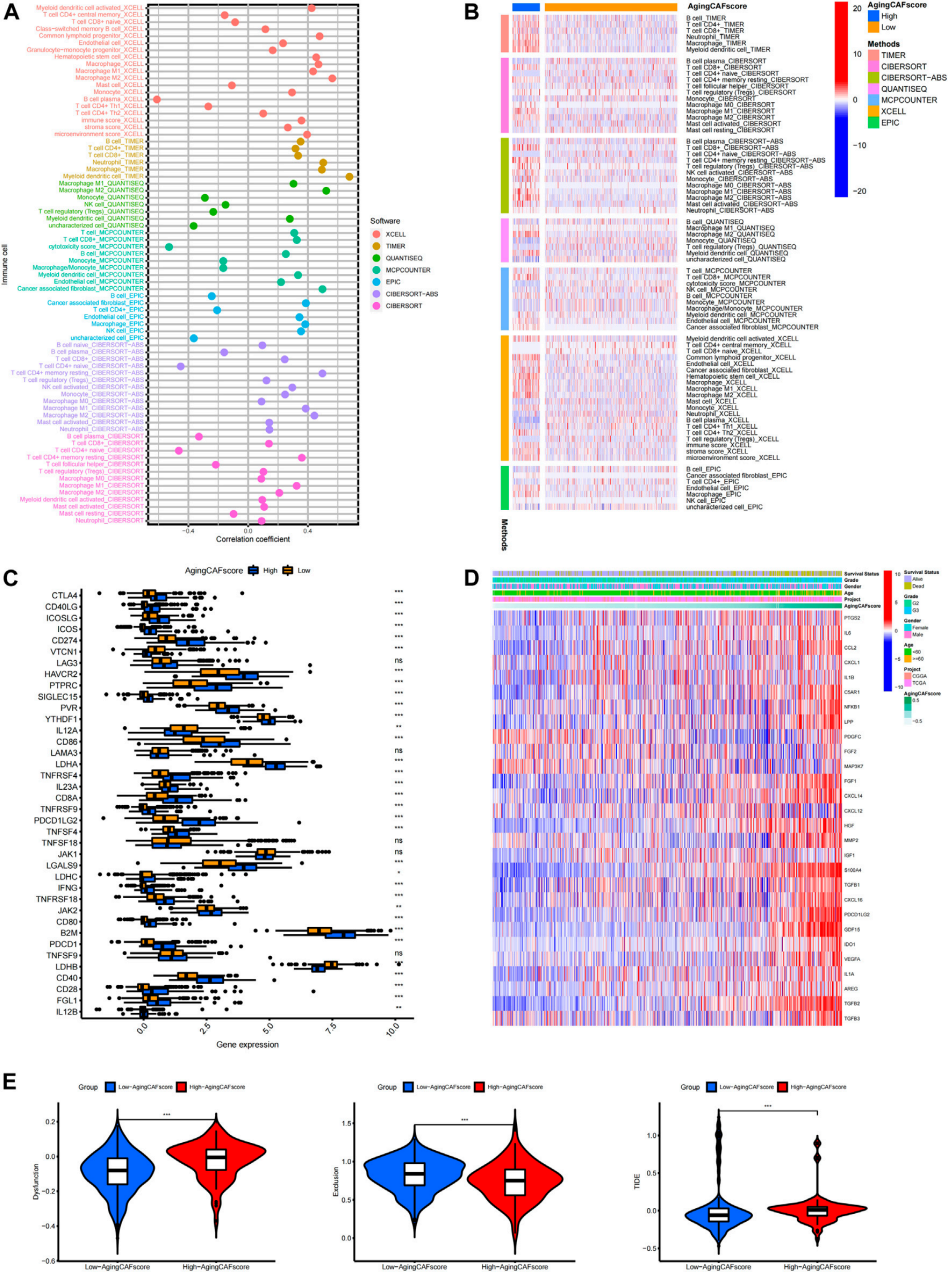
Exploration of the correlation between the aging CAF score and TME. **(A)** Correlation between the abundance of essential cells in the TME and aging CAF score. The cell types with *p*-values less than 0.05 were presented. **(B)** Heatmap showing the comparisons of the abundance of essential cells in the TME between low- and high-aging CAF score groups. The cell types with *p*-values less than 0.05 were presented. **(C)** Comparisons of the expression levels of immune checkpoints between the low- and high-aging CAF score groups. **(D)** Expression patterns of cytokines secreted by CAFs with increasing aging CAF scores. **(E)** Comparisons of TIDE-related scores between the two groups. * means *p* < 0.05, ** means *p* < 0.01, and ***means *p* < 0.001. CAF, cancer-associated fibroblast; TME, tumor microenvironment; TIDE, Tumor Immune Dysfunction and Exclusion.

The abundance of critical compositions in the TME calculated through multiple methods was involved in our study to extensively explore the correlation between the TME and aging CAF score (Figure 6A, *p* < 0.05). We found that immune score, stromal score, microenvironment score, and the abundance of stromal cells such as CAFs and epithelial cells were positively correlated with the aging CAF score. The abundance of macrophages, especially M2 macrophages which served as an anti-inflammatory and tumor-promoting phenotype (Yunna et al., 2020), was significantly associated with the aging CAF score. CD4^+^ T cells and CD8^+^ T cells were also positively correlated with the aging CAF score. Consistently, the abundance of M2 macrophages and CAFs significantly increased in the high-aging CAF score group (Figure 6B). Similar results were obtained by quantifying the TME components for all samples in the merged data *via* MCP counter, CIBERSORT algorithm, and ssGSEA method, in which CAFs, M2 macrophages, regulatory T cells (Treg), and myeloid-derived suppressor cells (MDSCs) increased in the high-aging CAF score group (Supplementary Figures S8A–C). We also found that most of the immune checkpoints were highly expressed in the high-aging CAF score group compared to the low-score group (Figure 6C). The expression levels of most of the genes involved in the negative regulation of the cancer-immunity cycle increased in the high-aging CAF score group (Supplementary Figures 8D,E), the related gene list was downloaded from the Tracking Tumor Immunophenotype website (http://biocc.hrbmu.edu.cn/). All these results suggested that the high-aging CAF score group tended to exhibit an immune-suppressive phenotype. Moreover, the expression levels of most of the cytokines secreted by CAFs upregulated with the increase in the aging CAF score (Figure 6D, Supplementary Figure S8F). Intriguingly, we found that some cytokines secreted by CAFs were also involved in senescence-associated secretory phenotype (SASP) factors which can be upregulated upon senescence, influence immune cell functions, and play tumor-promoting roles in the TME (Ruhland and Alspach, 2021), such as *IL6, AREG, CXCL12, TGFβ, VEGF,* and *CCL2.* These findings indicated that the aging CAF score developed by our study was genetically correlated with aging CAFs. The TIDE algorithm can be used to predict the potential response to immune checkpoint blockage treatment based on a comprehensive analysis of tumor immune dysfunction and exclusion mechanisms (Jiang et al., 2018). We detected that the dysfunction scores of the low-aging CAF score group were lower than those of the high-score group while the exclusion scores of the low-aging CAF score group were higher than those of the high-score group. Finally, we found that the low-aging CAF score group exhibited significantly lower TIDE scores than the high-score group, implying that patients in the low-aging CAF score group tended to benefit from immune checkpoint blockage treatment such as PD-1/PD-L1 blockage immunotherapy (all *p* values <0.001, Figure 6E).

In addition, the enrichment levels of the immune gene sets were quantified by single-sample gene set enrichment analysis (ssGSEA) based on the gene expression profiles of LGGs. Then, based on the ssGSEA scores of the immune cells, consensus clustering was performed to classify the LGG patients into different clusters, which were termed by immune subtypes (He et al., 2018). As shown in Supplementary Figure S9A, LGG samples were reasonable to be classified into three immune subtypes. We found that immune subtype B represented a subtype with high infiltration of immune cells, immune subtype C represented a subtype with low infiltration of immune cells, and immune subtype A represented a subtype with medium infiltration of immune cells (Supplementary Figure S9B). Furthermore, we explored the links between immune subtypes and the previously established model in our study. As shown in Supplementary Figures S9C,D, immune subtype B with high immunity showed the highest proportion of gene cluster A and the high-aging CAF score group while immune subtype C with low immunity showed the lowest proportion of gene cluster A and the high-aging CAF score group (*p* < 0.001).

### Exploration of genetic mutations for samples with low- and high-aging CAF scores

**FIGURE 7:**
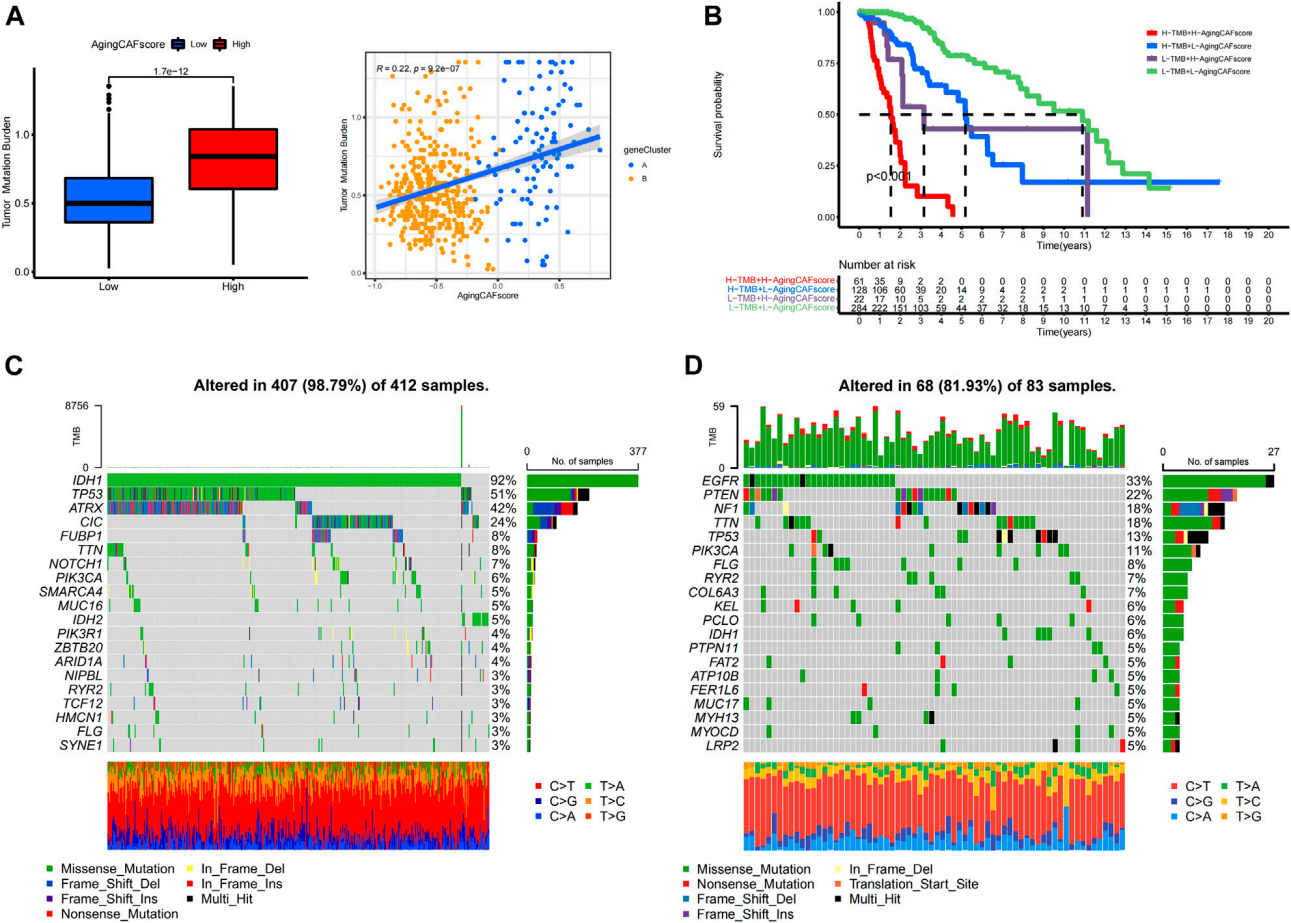
Exploration of genetic mutations for samples with low- and high-aging CAF scores. **(A)** Correlation between the TMB and aging CAF score. **(B)** Kaplan–Meier analysis of patients with different TMBs and aging CAF scores. **(C,D)** Top 20 genes with the highest mutation frequencies in the low- **(C)** and high- **(D)** aging CAF score groups. CAF, cancer-associated fibroblast; TMB, tumor mutation burden.

Previous studies demonstrated the underlying correlation between genetic alterations and the tumor immune microenvironment (Rooney et al., 2015). Thus, we further explored the features of genetic mutations for samples with low- and high-aging CAF scores. We found that TMBs were significantly lower in the low-aging CAF score group than in the high-score group (*p* = 1.7e-12) and the TMB was positively correlated with the aging CAF score (*R* = 0.22, *p* = 9.2e-7, Figure 7A). Moreover, the LGG patients with high TMBs and high-aging CAF scores received the shortest overall survival while the patients with low TMBs and low-aging CAF scores had the longest overall survival. The prognosis of patients with low TMBs and high-aging CAF scores was worse than those of patients with high TMBs and low-aging CAF scores, indicating that the aging CAF score served as an independent prognostic factor independent of the TMB (Figure 7B). Finally, we identified the top 20 genes with the highest mutation frequencies in the low- and high-aging CAF score groups (Figures 7C,D). *IDH1*, *TP53,* and *ATRX* represented the top three frequently mutated genes in the low-aging CAF score group while *EGFR*, *PTEN,* and *NF1* presented the highest mutation frequencies in the high-aging CAF score group.

### Validation of aging CAF-related genes in external datasets

**FIGURE 8:**
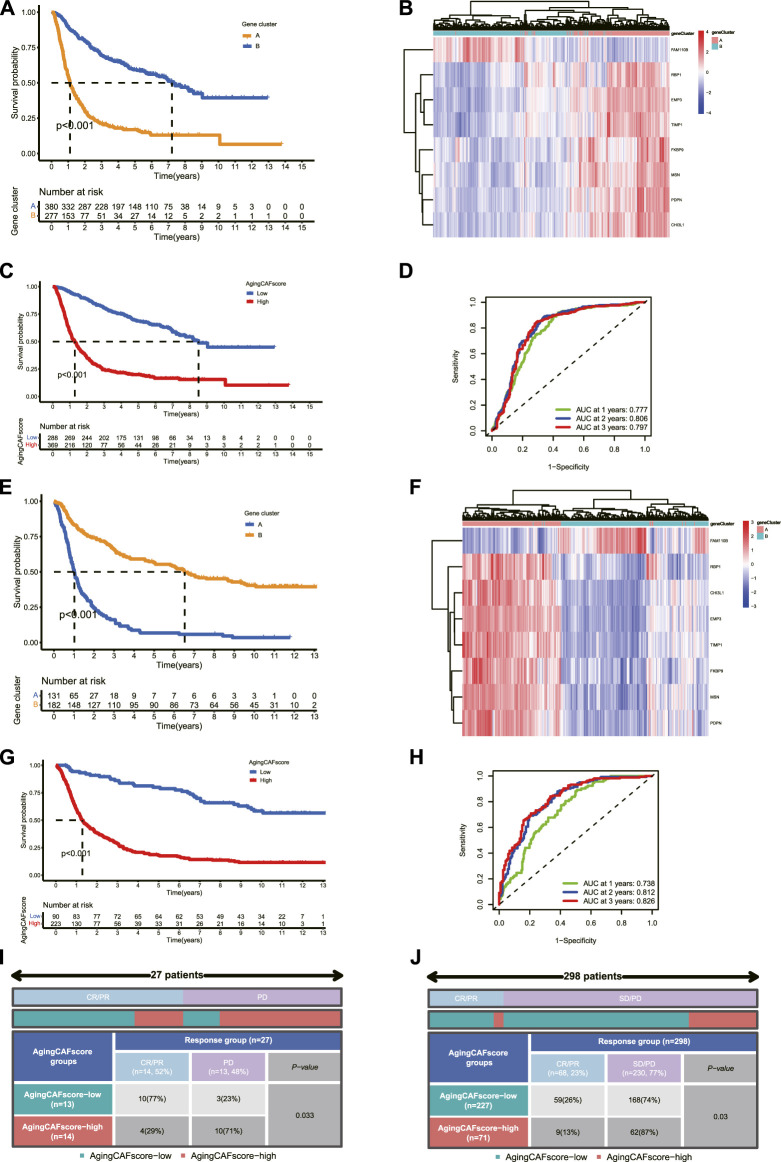
Validation of aging CAF-related genes in external datasets. **(A)** Comparison of the overall survival between gene clusters A and B in the validation cohort (dataset ID: mRNAseq_693). **(B)** Unsupervised clustering for samples based on the expression of eight featured ACAFRGs in the validation cohort (dataset ID: mRNAseq_693). **(C)** Comparison of the overall survival between the low- and high-aging CAF score groups in the validation cohort (dataset ID: mRNAseq_693). **(D)** Time-dependent ROC curves of the aging CAF score in the validation cohort (dataset ID: mRNAseq_693). **(E–H)** Similar results were obtained in another validation cohort (dataset ID: mRNAseq_325). **(I)** Comparison of the response to PD-1 immune checkpoint blockage treatment between the low- and high-aging CAF score groups in the GSE78220 cohort. **(J)** Comparison of the response to PD-L1 immune checkpoint blockage treatment between the low- and high-aging CAF score groups in the IMvigor210 cohort. CAF, cancer-associated fibroblast; ACAFRGs, aging cancer-associated fibroblast related genes; ROC, receiver operating characteristic; AUC, area under curve; CR/PR, complete remission/partial remission; PD/SD, progressed disease/stable disease.

Two independent cohorts were employed to verify the aging CAF-related genes. First, two distinct aging CAF-related gene clusters were identified in the validation cohort (dataset ID: mRNAseq_693) based on the expression profiles of aging CAF-related genes by the consensus clustering method (Supplementary Figure S10A). Consistent with the aforementioned results, the prognosis of gene cluster A was worse than that of gene cluster B (Figure 8A). Moreover, we found that the expression levels of the eight featured genes for discriminating the two gene clusters substantially differed between gene clusters A and B in the validation cohort (Supplementary Figure S10B). The ROC curves demonstrated the high accuracy of the eight featured genes for discriminating the two gene clusters (Supplementary Figure S10C). Unsupervised clustering for glioma samples in the validation cohort demonstrated that samples can be easily discriminated against based on the expression of the eight featured ACAFRGs (Figure 8B). A nomogram model was also built to predict the probability of gene cluster A based on the expression of these featured genes (Supplementary Figure S10D). The calibration curve, DCA curve, and clinical impact curve confirmed the robust performance of the nomogram model (Supplementary Figures S6E–G). Finally, samples in the validation cohort were assigned with specific aging CAF scores based on a similar method, followed by which samples were separated into low- and high-aging CAF score groups. The prognosis of the high-aging CAF score group was worse than those of the low-score group (Figure 8C). The ROC curves for predicting 1, 2, and 3-year overall survival further verified the accuracy of the aging CAF score (Figure 8D). In addition, similar results were obtained in another validation cohort (dataset ID: mRNAseq_325, Figures 8E–H, Supplementary Figure S11).

We also verified the performance of the aging CAF score in predicting the response to immune checkpoint blockage treatment in two independent cohorts. More patients were found to get favorable responses in the low-aging CAF score group compared to the high-score group (*p* = 0.033 in the GSE78220 cohort and *p* = 0.03 in the IMvigor210 cohort, Figures 8I,J).

### Validation of the featured ACAFRGs at the protein level

**FIGURE 9:**
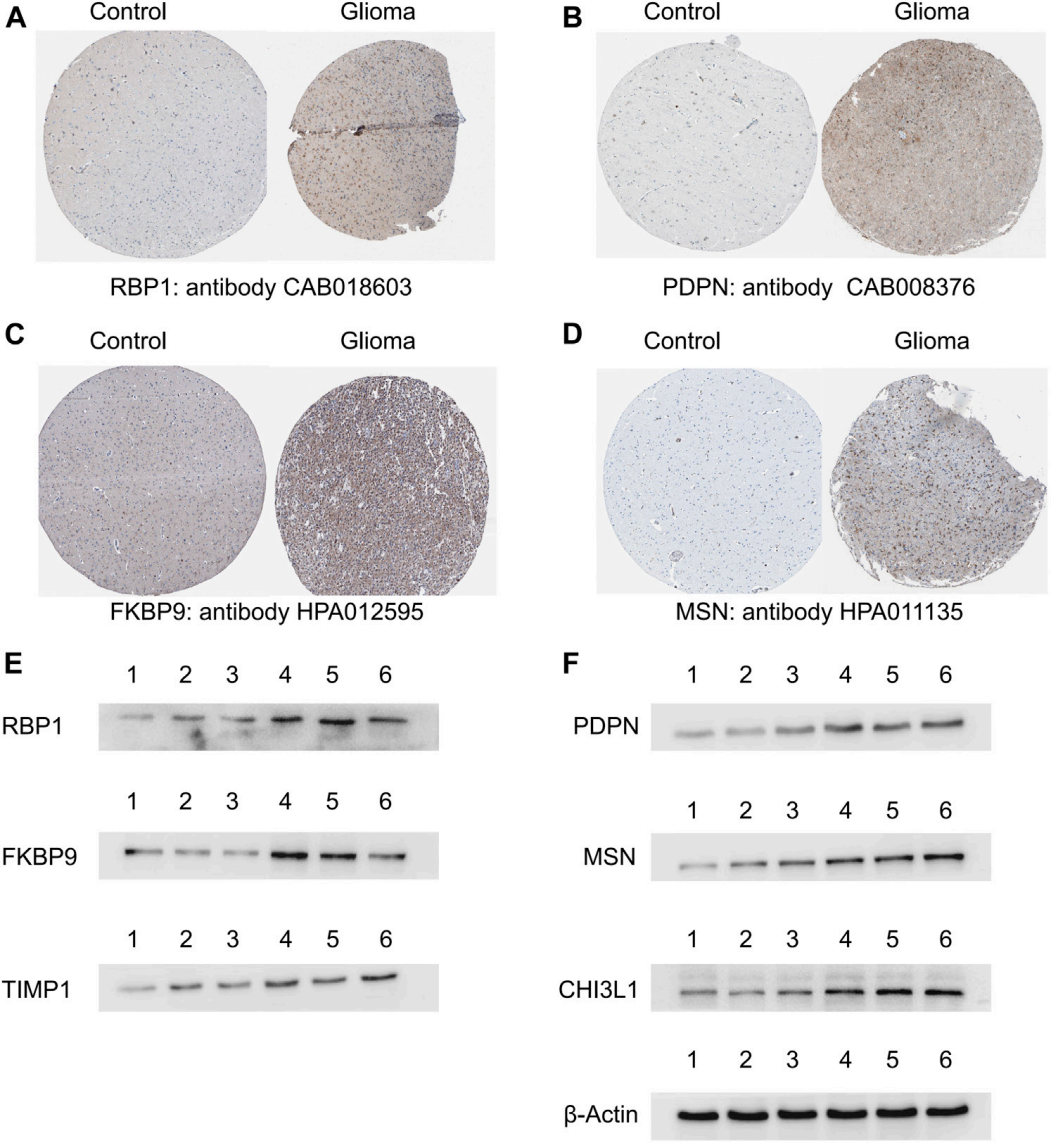
Validation of the featured ACAFRGs at the protein level. **(A–D)** Differential expression patterns of the featured genes between normal brain tissues and glioma tissues which were identified in immunohistochemistry staining on the Human Protein Atlas website. **(E)** Identification of the featured genes by Western blotting, in which lane 1 represents normal brain tissues, lanes 2 and 3 represent grade II glioma tissues, and lanes 4, 5, and 6 represent grade III glioma tissues. ACAFRGs, aging cancer-associated fibroblast related genes; Control: normal brain tissue.

A total of four ACAFRGs, namely, *RBP1, PDPN, FKBP9,* and *MSN* were randomly selected from the featured ACAFRGs. We found differential expression patterns of the aforementioned genes between normal and glioma tissues in immunohistochemistry staining on the Human Protein Atlas website (Figures 9A–D). Western blotting confirmed the high expression levels of six ACAFRGs in glioma tissues at the protein level (Figure 9E). As shown in Supplementary Figure S12, for the six molecules, we found that the optical density of G3 glioma samples was significantly higher than those of the normal brain samples. Although we detected that the six molecules were upregulated in the G2 glioma samples compared to the normal brain samples (Figure 9E), the statistical analysis of the optical density of the bands demonstrated no significant difference between them except PDPN and RBP1.

## Discussions

As the most common stromal component in the TME, CAFs have been drawing increasing attention in cancer research for their indispensable roles in tumor initiation and progression. Previous studies have revealed that CAFs have context-dependent functions, harboring both tumor-promoting and tumor-suppressive roles (Chen et al., 2021). The significant impact of CAFs on the regulation of anti-tumor immunity makes it possible to predict the immunotherapy response based on CAF-related biomarkers (Miyai et al., 2022). Moreover, considering that cancer has been previously regarded as a disease of aging, CAFs are demonstrated to be particularly susceptible to aging-related impact in the context of tumor development (Yasuda et al., 2021a). Therefore, studies focusing on aging CAFs may provide a new direction for exploring biomarkers by drawing implications in predicting prognosis and immunotherapy in gliomas. In our study, we identified ACAFRGs in LGGs by the WGCNA method, based on which LGG samples were classified into two aging CAF-related gene clusters with distinct prognosis and characteristics of the TME. Machine learning algorithms were used to screen out the eight featured ACAFRGs to characterize two aging CAF-related gene clusters, and a nomogram model was constructed to predict the probability of gene cluster A for each LGG sample. Then, a powerful aging CAF scoring system was developed to predict the prognosis and response to immune checkpoint blockage therapy in the current research. Finally, the ACAFRGs were verified in two glioma-related external datasets. The performance of the aging CAF score in predicting the immunotherapy response was further validated in two independent cohorts with the information on immune checkpoint blockage treatment. We also confirmed the expression of ACAFRGs at the protein level in glioma tissues.

CAFs are a critical component in the stroma of the TME with a variety of functions, including generating and remodeling extracellular matrix components and complex interactions with tumor cells and other cell types in the TME (Sahai et al., 2020). Epigenetic alterations of CAFs enable the production and release of multiple cytokines, chemokines, exosomes, and metabolites, which impacts cancer progression, regulation of anti-tumor immunity, and metabolism (Chen et al., 2021). Based on the distinct transcriptome, single-cell RNA sequencing analysis has identified several subpopulations of CAFs with different functions, which has increased our understanding of the high heterogeneity of CAFs (Kieffer et al., 2020). Similar to the opposing effects of CAFs on tumor cells (tumor-permissive and tumor-suppressive effects), aging CAFs have also been demonstrated to exhibit both pro- and anti-tumorigenic activity (Ruhland and Alspach, 2021). Nevertheless, the substantial roles of aging CAFs in tumor promotion have always been underscored in recent years. Researchers have reported the dynamic evolving epigenetic changes of CAFs in the course of aging. Aging CAFs facilitate tumor progression mainly by the secretion of SASP factors which can cause chronic inflammation, promote angiogenesis, and enhance immunosuppressive activity (Yasuda et al., 2021a). However, it is important to note that current studies focusing on aging CAFs are facing the challenge of identifying both the aging status and the cell type of CAFs. Fibroblasts can be determined only by the absence of markers which define epithelial cells, endothelial cells, and immune cells (Sahai et al., 2020). In our study, we determined a total of 463 ACAFRGs which were significantly correlated with aging CAFs in LGG samples, such as *CDKN2B, CCL4, CCL19,* and *ISLR*. The *CDKN2B* (cyclin-dependent kinase inhibitor 2B) gene locates at exon 1 of *CDKN2B-AS1,* and the encoded protein serves as a regulator in cell cycle G1 progression by interacting with CDK kinases (Sibin et al., 2016). *CDKN2B* is found to be highly differentially expressed in aged individuals (Sebastiani et al., 2021). Considering that cellular senescence has previously been defined as a state of permanent cell cycle arrest, as a cell cycle arrest gene, *CDKN2B* has been reported to contribute to extracellular matrix deposition and cellular senescence (Rathi et al., 2020). CCL family members are involved in the chemokines and cytokines which can be strongly expressed by CAFs to enhance the pro-tumorigenic activity of myeloid cells (Monteran and Erez, 2019). Meflin (*ISLR*) is defined as a new cell surface marker for cancer-restraining CAFs in pancreatic and colon cancers. Tumor-suppressive roles of meflin-positive CAFs have been proposed, which are mediated by the regulation of collagen structures and bone morphogenetic protein (BMP) signaling in the TME (Takahashi et al., 2021). Meflin has been also reported to correlate with favorable prognosis and therapeutic response to immune checkpoint blockage treatment in patients with non-small cell lung cancer (NSCLC) (Miyai et al., 2022). Alternatively, meflin is determined as an unfavorable gene with a predictive value in patients with colon adenocarcinoma (Wang et al., 2021). Consistently, univariate Cox regression analysis revealed that meflin (*ISLR*) served as an unfavorable gene in LGGs in our research.

A total of 400 ACAFRGs with prognostic values were used to segregate LGG samples into two aging CAF-related gene clusters. We found that worse survival was associated with gene cluster A which was characterized by more infiltrating immune cells and fibroblasts in the TME, compared to gene cluster B. A total of eight featured ACAFRGs were determined to discriminate the two gene clusters, namely, *FAM110B, RBP1, FKBP9, MSN, PDPN, TIMP1, EMP3, and CHI3L1*, based on which a nomogram with robust performance was constructed to predict the probability to be grouped into gene cluster A for each glioma patient. In addition, each gene involved in the eight featured ACAFRGs exhibited high accuracy to characterize the gene clusters. For example, as a member of the FAM110 family (family with sequence similarity 110), *FAM110B* with an AUC value of 0.934 was downregulated in gene cluster A and the high-aging CAF score group (Supplementary Figure S4, Figure 3K, Figure 4I). Previous studies demonstrated that *FAM110B* participated in the regulation of the cell cycle and predicted favorable prognosis in NSCLC (Xie et al., 2020). In agreement with this study, *FAM110B* was found to be involved in the favorable gene set which was used to calculate the aging CAF score. As an unfavorable gene involved in ACAFRGs, *CHI3L1* was highly expressed in gene cluster A and the high-aging CAF score group (Figure 3K, Figure 4H). Consistent with our results, *CHI3L1* has been reported to be associated with poor prognosis in hepatocellular carcinoma (Wang et al., 2022). A recent study showed that *CHI3L1* was significantly correlated with severe state and adverse prognosis for COVID-19 patients (Kimura et al., 2021). Intriguingly, *CHI3L1* has been shown to positively regulate the PD-1/PD-L1 axis and other immune checkpoint molecules, potentially implying its impact on immunotherapy response (Ma et al., 2021). It is important to emphasize that the high-aging CAF score group has been found to receive less response to immune checkpoint blockage therapy in the current research, which might be attributed to the aforementioned mechanism.

Based on the expression profiles of unfavorable and favorable ACAFRGs, we constructed a novel aging CAF scoring system. We detected that the high-aging CAF score predicted poor prognosis and a less favorable response to immune checkpoint blockage therapy in LGGs. The powerful performance of the aging CAF score was further verified in external cohorts. In this study, our analysis culminated in several important points: 1) as shown in Supplementary Figure S13, the aging CAF score was positively correlated with age (R = 0.17, *p* = 1.7e-5) and the age values of the high-aging CAF score group were significantly higher than those of the low-score group (*p* = 1.9e-12); 2) the aging CAF score was shown to be positively associated with the abundance of CAFs within the TME, as shown in Figure 6A; 3) the SASP factors and cytokines secreted by CAFs were robustly upregulated with the increasing aging CAF score. All these findings suggested that, except for the predictive performance, the aging CAF score developed in our study may serve as an indicator to quantify the abundance of aging CAFs in the TME. To some extent, the high aging CAF score predicted a poor prognosis and indicated more abundance of aging CAFs in the TME, which was consistent with the widely accepted concept that aging CAFs contributed to the proliferation and invasion of the surrounding cancer cells (Yasuda et al., 2021a). In addition, previous studies have revealed that aging CAFs promote the recruitment of M2 macrophages to enhance the immunosuppressive phenotype (Ruhland et al., 2016). We found that well-defined immune cells negatively regulating immune responses such as regulatory T cells (Tregs), M2 macrophages, and MDSCs presented high abundance in the high-aging CAF score group accompanied by the high expression of genes negatively regulating anti-tumor immunity (Figure 6B, Supplementary Figure S8B–E). This indicated that the high-aging CAF score group had an immunosuppressive phenotype, which was in accordance with the current point that aging CAFs acted as a major driver of immunosuppression within the TME (Ruhland and Alspach, 2021). To confirm the outstanding performance of the aging CAF score, we compared the risk score from multivariate Cox regression analysis based on ACAFRGs with it. First, we calculated the risk score for each LGG sample based on the expression profiles of prognostic ACAFRGs by using multivariate Cox regression analysis method. The critical genes involved in the construction of the risk score and the corresponding coefficients are listed in Supplementary Table S4. Similarly, LGG samples were divided into high- and low-risk score groups. As shown in Supplementary Figures S14A,B, LGG patients in the high-risk score group had a worse prognosis than those in the low-risk score group (*p* < 0.001) and the univariate/multivariate Cox regression analysis indicated that the risk score was significantly correlated with prognosis and served as an independent prognostic factor (all *p* values < 0.001). However, the ROC curves revealed that the accuracy of the aging CAF score was higher than those of the risk score for predicting the prognosis (Supplementary Figures S14C–E). For example, the AUC value of the aging CAF score for predicting the 2-year overall survival was 0.802 while the AUC value of the risk score was 0.776. Similarly, the results of DCA showed that the aging CAF score was a more powerful predictor than the risk score. Moreover, as shown in Supplementary Figure S14F, the C-index value of the aging CAF score group (0.942) was higher than those of the risk score group (0.913). Similar to the aging CAF score, the risk score can also predict the characteristics of the TME (Supplementary Figure S14G), immunotherapy response (Supplementary Figure S14H), aging CAF-related gene clusters (Supplementary Figure S14I), and tumor mutation burden (Supplementary Figure S14J). However, the risk score cannot predict the immunotherapy response in the validation cohorts (*p* = 0.853 in GSE78220, *p* = 0.077 in the IMvigor210 cohort, Supplementary Figures S14K,L). The robust capacity of the aging CAF score for predicting the immunotherapy response has been verified in the aforementioned external datasets in our study. Taking all these results into consideration, we believe that the aging CAF score in our study has a more powerful potential to predict prognosis and immunotherapy response than the risk score from multivariate Cox regression analysis.

Moreover, our results indicated that the high-aging CAF score group bore more TMBs than the low-score group (Figure 7A). It is to be noted that the top three genes with the highest mutation frequencies in the high-aging CAF score group were *EGFR*, *PTEN,* and *NF1*. The mutation frequency of *EGFR* in GBMs has been shown to be higher than those in LGGs. *EGFR* mutation has been reported to be an independent predictor of the prognosis in all grades of gliomas (Saadeh et al., 2017). *PTEN* mutation has also been found to be significantly associated with reduced survival in gliomas (Zhang et al., 2021). Wang et al. demonstrated that *NF1* was more frequently mutated in GBMs compared to LGGs and has been used to define the mesenchymal subtype of GBMs (Verhaak et al., 2010). In contrast, *IDH1* exhibited the highest mutation frequency in the low-aging CAF score group, which was consistent with the well-identified concept that *IDH* mutation was associated with better prognosis in gliomas (Turkalp et al., 2014). It seemed that LGGs in the high-aging CAF score group may represent a subgroup similar to GBMs from the point of genetic variation.

Overall, in our study, we determined a series of ACAFRGs in LGGs, based on which a robust aging CAF scoring system was developed to predict the prognosis and immunotherapy response. Our findings may provide new targets for therapeutics and contribute to future exploration focusing on aging CAFs.

## Data Availability

Publicly available datasets were analyzed in this study. We declare that the datasets supporting the findings of this study are available in TCGA database (https://portal.gdc.cancer.gov/), CGGA database(http://cgga.org.cn/index.jsp), and GEO database (https://www.ncbi.nlm.nih.gov/geo/).
