# Supplementary material for: Characterization of aging cancer-associated fibroblasts draws implications in prognosis and immunotherapy response in low-grade gliomas
Source: Front Genet. 2022 Aug 24;13:897083. doi: 10.3389/fgene.2022.897083 (PMC9449154; doi:10.3389/fgene.2022.897083)
Supplement: Supplementary file 1 [file DataSheet7.PDF]

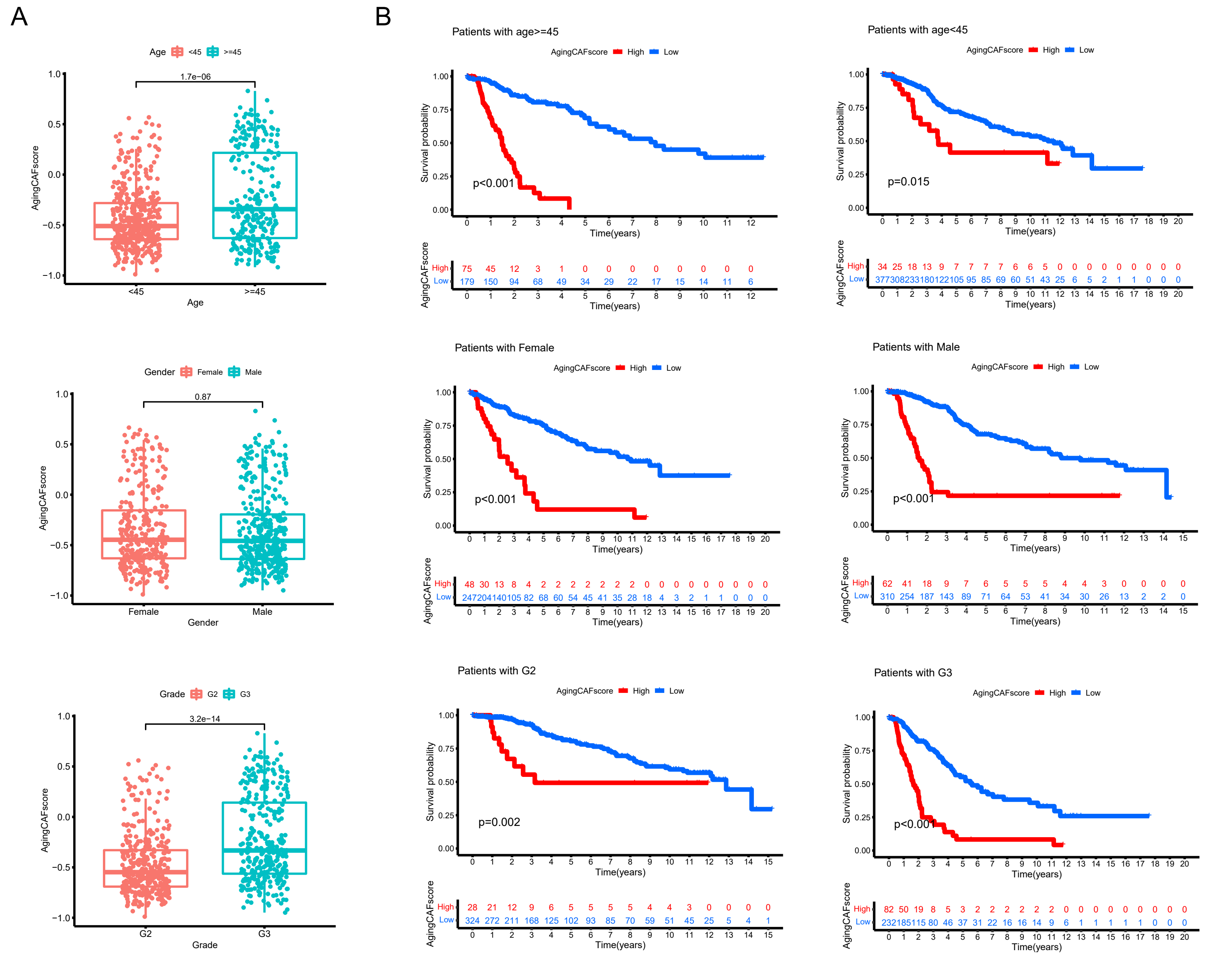

Supplementary figure 7. (A) Comparisons of aging CAF scores between LGG patients with different clinical features. (B) Kaplan–Meier survival analysis between low and high aging CAF score groups with different clinical features. CAF, cancer associated fibroblast.
