# Supplementary material for: Characterization of aging cancer-associated fibroblasts draws implications in prognosis and immunotherapy response in low-grade gliomas
Source: Front Genet. 2022 Aug 24;13:897083. doi: 10.3389/fgene.2022.897083 (PMC9449154; doi:10.3389/fgene.2022.897083)
Supplement: Supplementary file 2 [file DataSheet13.PDF]

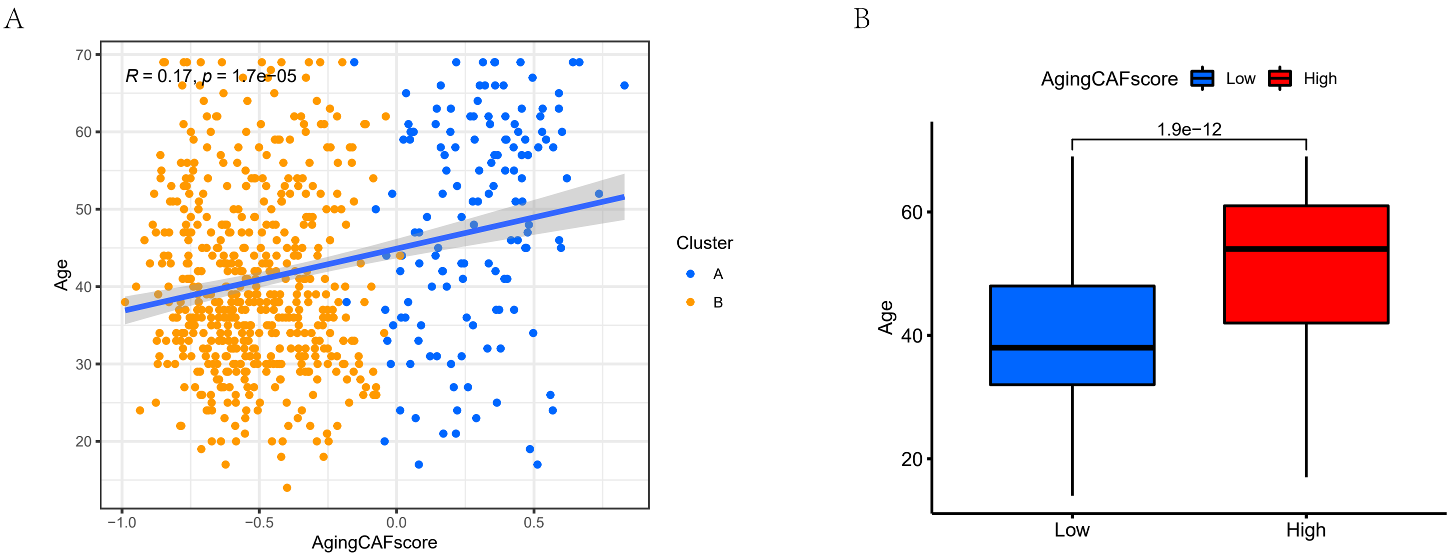

Supplementary figure 13. (A) The correlation between aging CAF score and age. (B) Comparison of age bwtween the low and high-aging CAF score groups.
