# Supplementary material for: Characterization of aging cancer-associated fibroblasts draws implications in prognosis and immunotherapy response in low-grade gliomas
Source: Front Genet. 2022 Aug 24;13:897083. doi: 10.3389/fgene.2022.897083 (PMC9449154; doi:10.3389/fgene.2022.897083)
Supplement: Supplementary file 3 [file DataSheet2.PDF]

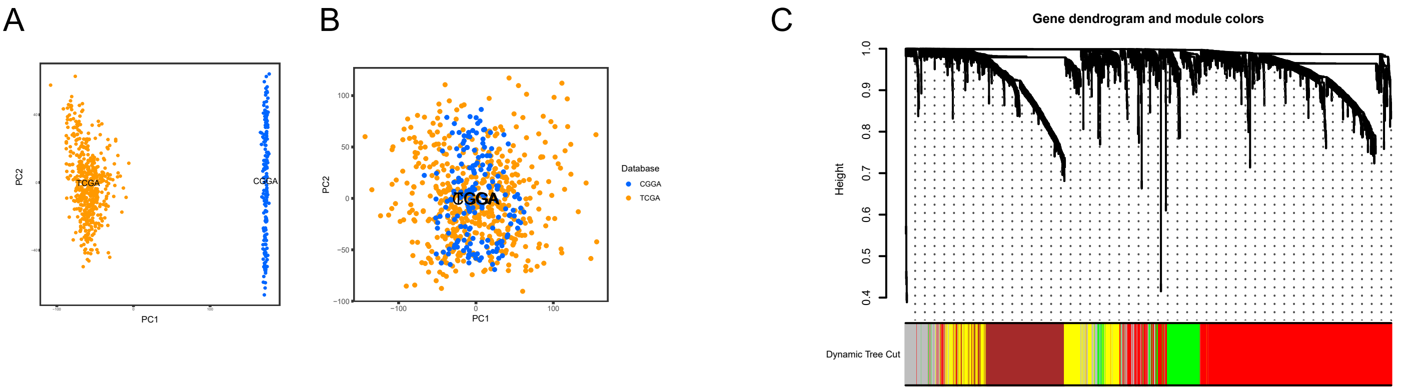

Supplementary figure 2. (A, B) Based on the total gene expression profiles, two-dimensional PCA cluster plot for samples in TCGA and CGGA databases before (A) and after (B) batch effect correction. The labels for databases were located at the center of the distribution of samples. (C) Cluster dendrogram of genes in different modules in the co-expression network.
