# Supplementary material for: Characterization of aging cancer-associated fibroblasts draws implications in prognosis and immunotherapy response in low-grade gliomas
Source: Front Genet. 2022 Aug 24;13:897083. doi: 10.3389/fgene.2022.897083 (PMC9449154; doi:10.3389/fgene.2022.897083)
Supplement: Supplementary file 4 [file DataSheet4.PDF]

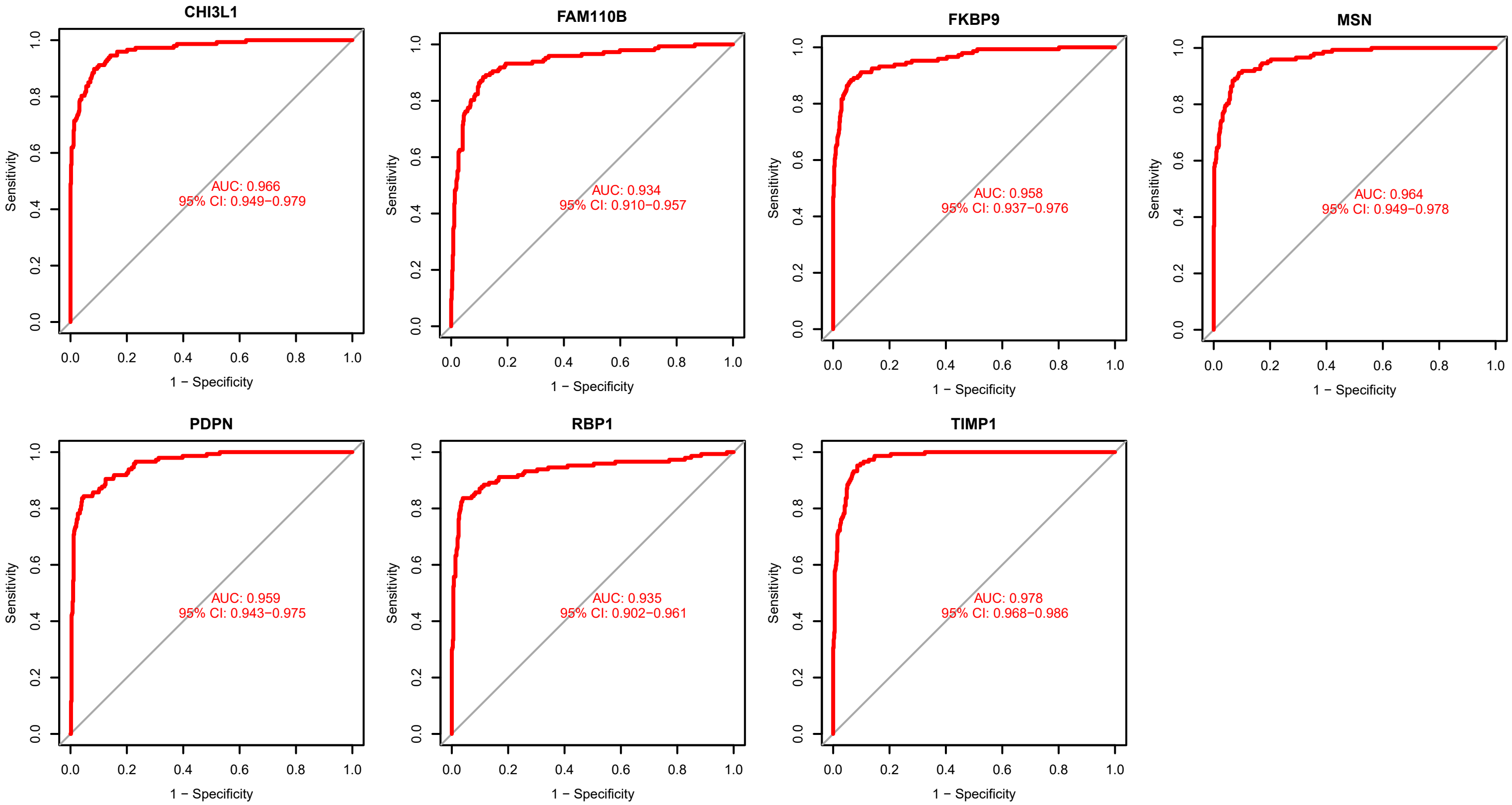

Supplementary figure 4. ROC curves demonstrating the accuracy of the featured genes for discriminating two aging CAF related gene clusters. CAF, cancer associated fibroblast; ROC, receiver operating characteristic; AUC, area under curves.
