# Supplementary material for: Characterization of aging cancer-associated fibroblasts draws implications in prognosis and immunotherapy response in low-grade gliomas
Source: Front Genet. 2022 Aug 24;13:897083. doi: 10.3389/fgene.2022.897083 (PMC9449154; doi:10.3389/fgene.2022.897083)
Supplement: Supplementary file 5 [file DataSheet6.PDF]

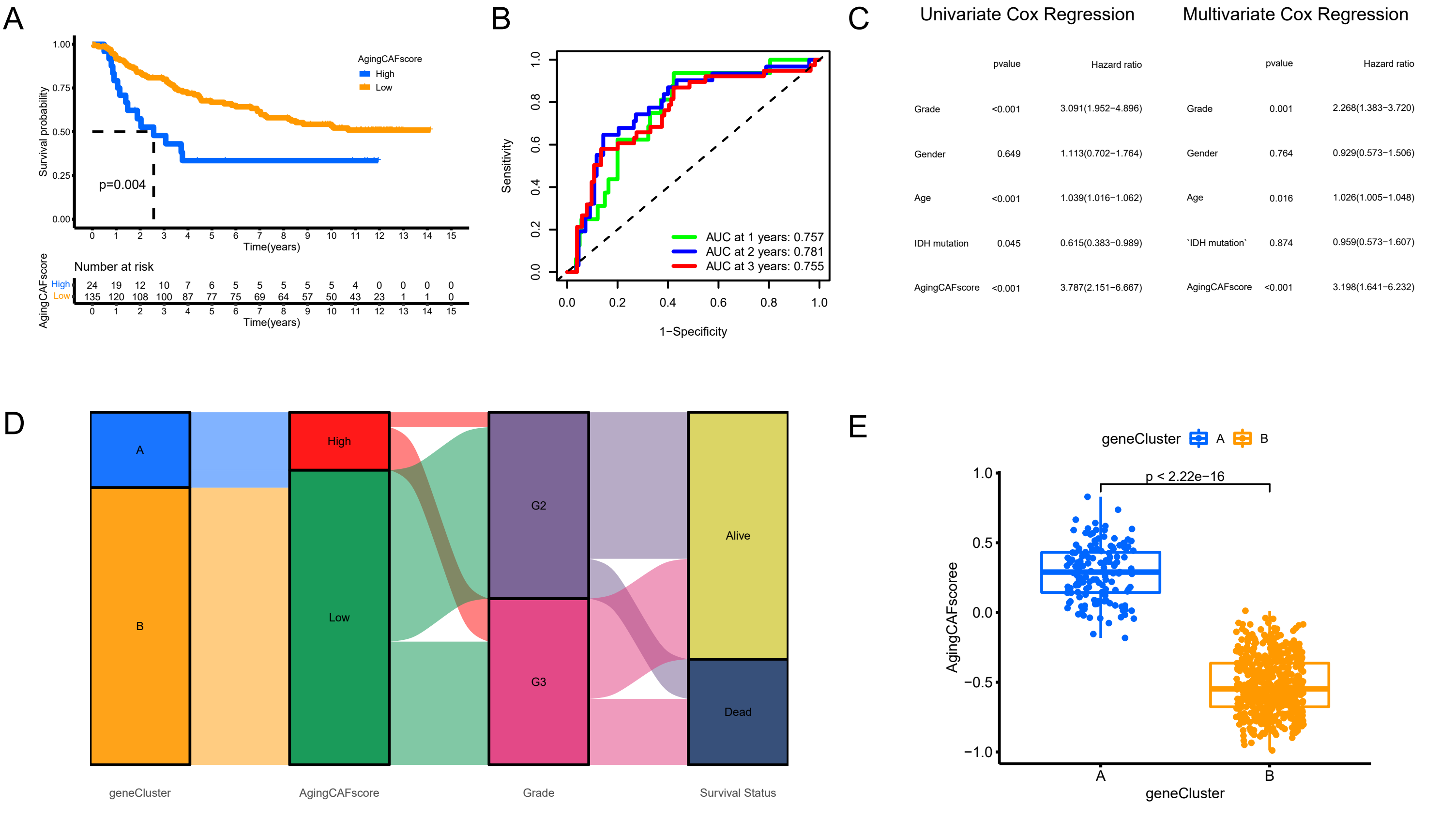

Supplementary figure 6. (A) Kaplan − Meier survival analysis between the low and high-aging CAF score groups in CGGA cohort. (B) Time-dependent ROC curves of aging CAF score in CGGA cohort. (C) Univariate/multivariate cox regression analysis of aging CAF score in CGGA cohort. (D) Alluvial diagram of gene clusters, aging CAF score groups, grade and survival status. (E) Comparisons of aging CAF scores between gene cluster A and B.
