# Supplementary material for: Characterization of aging cancer-associated fibroblasts draws implications in prognosis and immunotherapy response in low-grade gliomas
Source: Front Genet. 2022 Aug 24;13:897083. doi: 10.3389/fgene.2022.897083 (PMC9449154; doi:10.3389/fgene.2022.897083)
Supplement: Supplementary file 6 [file DataSheet14.PDF]

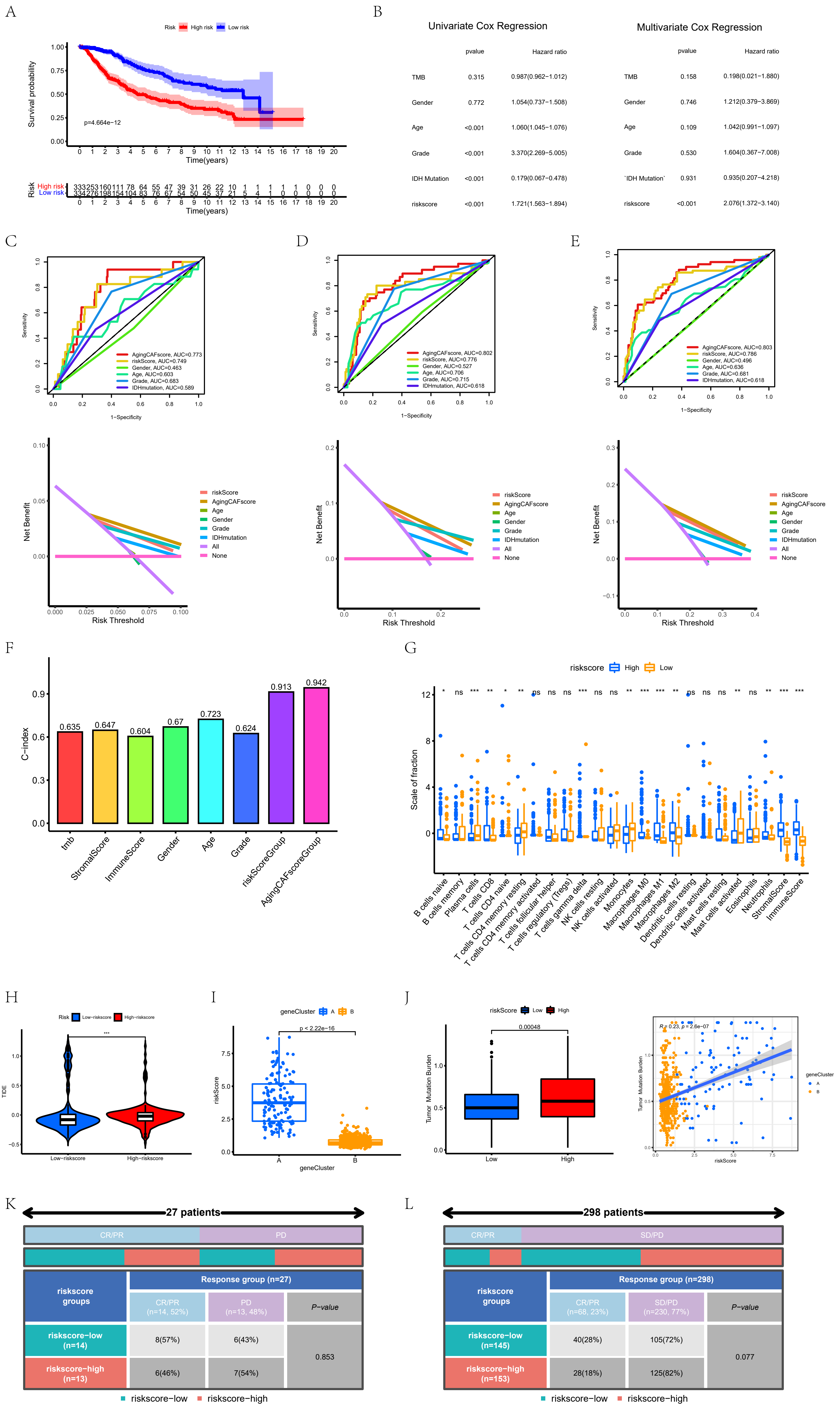

Supplementary figure 14. (A)Kaplan − Meier survival analysis between the low and high risk score groups. (B)Univariate/multiva cox regression analysis of risk score. (C) The ROC curves of risk score for predicting the 1 -year overall survival and DCA of risk score for predicting the 1-year prognosis. (D,E) Similar results were obtained in for predicting 2 (D) and 3 (E)-year overall survival. (F) The values of C-index for the risk score group and aging CAF score group for predicting prognosis. (G) Comparisons of TME components between the high and low risk score groups by using CIBERSORT algorithm. (H) Comparisons of TIDE related scores between the two risk score groups. (I) Comparison of risk scores between gene cluster A and B. (J) The correlation between TMB and risk score. (K)Comparison of the response to PD-1 immune checkpoint blockage treatment between the low and high-risk score group in GSE78220 cohort. (L) Comparison of the response to PD-L1 immune checkpoint blockage treatment between the low and high-risk score group in IMvigor210 cohort. ROC, receiver operating characteristic; AUC, area under curves; DCA, decision curve analysis; C-index, consistency index;CAF, cancer associated fibroblast; TME, tumor microenvironment; TIDE, Tumor Immune Dysfunction and Exclusion. CR/PR, complete remission/ partial remission; PD/SD, progressed disease/stable disease. * means $p < 0.05$, ** means $p < 0.01$, and ***means $p < 0.001$.
