# Supplementary material for: Characterization of aging cancer-associated fibroblasts draws implications in prognosis and immunotherapy response in low-grade gliomas
Source: Front Genet. 2022 Aug 24;13:897083. doi: 10.3389/fgene.2022.897083 (PMC9449154; doi:10.3389/fgene.2022.897083)
Supplement: Supplementary file 7 [file DataSheet9.PDF]

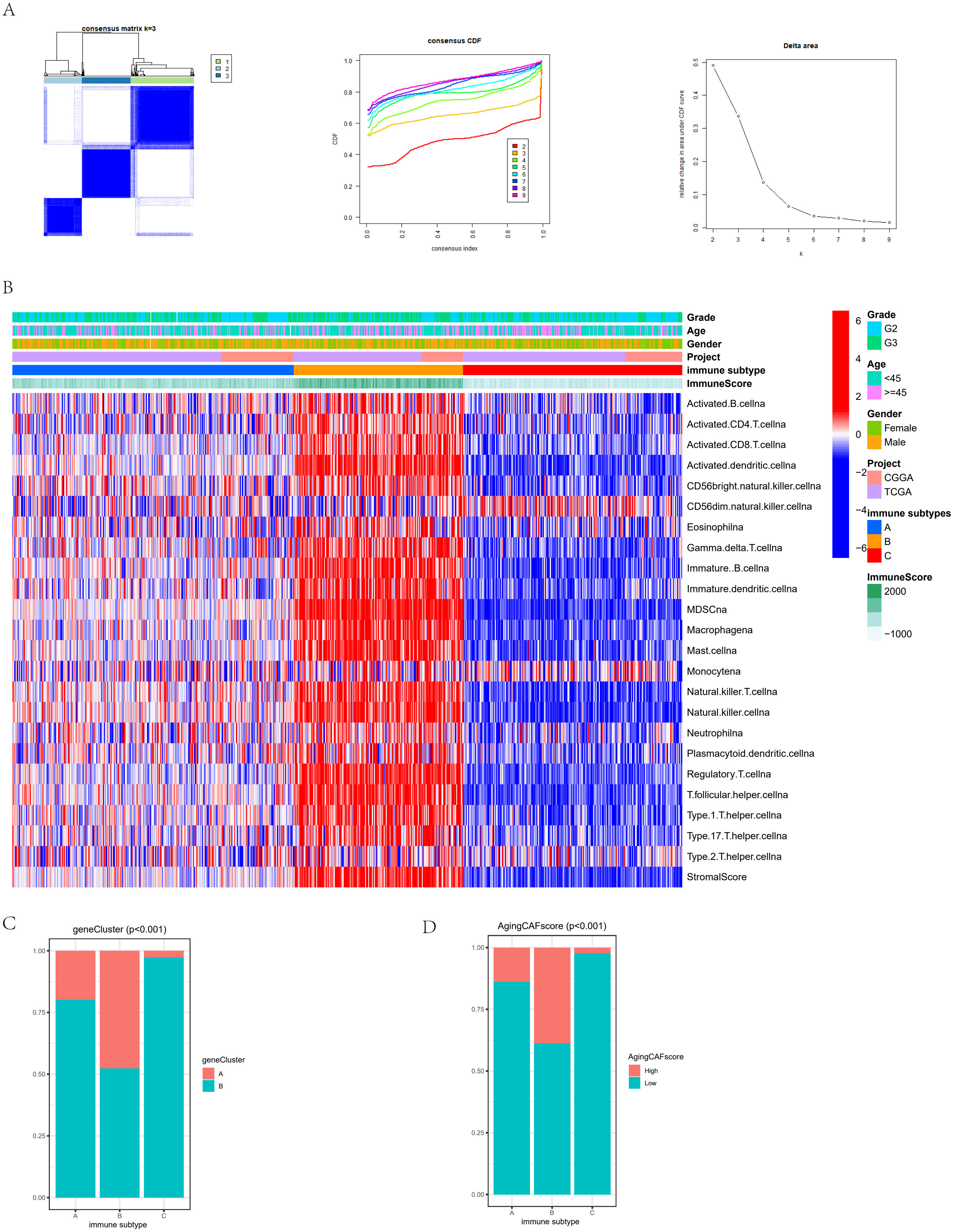

Supplementary figure 9. (A) Consensus clustering was performed to classify the LGG patients into different immune subtypes. (B) Heatmap showing the patterns of the infiltration of immune cells for three immune subtypes. (C) The correlation between aging CAF related gene clusters and immune subtypes. (D) The correlation between aging CAF score groups and immune subtypes.
