# Supplementary material for: Characterization of aging cancer-associated fibroblasts draws implications in prognosis and immunotherapy response in low-grade gliomas
Source: Front Genet. 2022 Aug 24;13:897083. doi: 10.3389/fgene.2022.897083 (PMC9449154; doi:10.3389/fgene.2022.897083)
Supplement: Supplementary file 8 [file Table1.DOCX]

**Supplementary table 1. Clinicopathological features of LGG patients in TCGA database**

| Features |  | Total | High-aging  CAFscore | Low-aging  CAFscore |
| --- | --- | --- | --- | --- |
| Age | <45 | 300(59.06%) | 20(23.26%) | 280(66.35%) |
|  | >=45 | 208(40.94%) | 66(76.74%) | 142(33.65%) |
| Grade | G2 | 246(48.52%) | 19(22.09%) | 227(53.92%) |
|  | G3 | 261(51.48%) | 67(77.91%) | 194(46.08%) |
| Histologic  Type | Astrocytoma | 192(37.8%) | 57(66.28%) | 135(31.99%) |
|  | Oligoastrocytoma | 128(25.2%) | 14(16.28%) | 114(27.01%) |
|  | Oligodendroglioma | 188(37.01%) | 15(17.44%) | 173(41%) |
| IDH1  Mutation | Mutant | 91(72.8%) | 5(20%) | 86(86%) |
|  | Wildtype | 34(27.2%) | 20(80%) | 14(14%) |
| Gender | Female | 508(100%) | 86(100%) | 422(100%) |
|  | Male | 226(44.49%) | 40(46.51%) | 186(44.08%) |
| Therapy Outcome | Complete Remission/Response | 282(55.51%) | 46(53.49%) | 236(55.92%) |
|  | Partial Remission/Response | 83(35.93%) | 9(23.68%) | 74(38.34%) |
|  | Progressive Disease | 50(21.65%) | 4(10.53%) | 46(23.83%) |
|  | Stable Disease | 39(16.88%) | 17(44.74%) | 22(11.4%) |
