# Supplementary material for: Characterization of aging cancer-associated fibroblasts draws implications in prognosis and immunotherapy response in low-grade gliomas
Source: Front Genet. 2022 Aug 24;13:897083. doi: 10.3389/fgene.2022.897083 (PMC9449154; doi:10.3389/fgene.2022.897083)
Supplement: Supplementary file 9 [file DataSheet11.PDF]

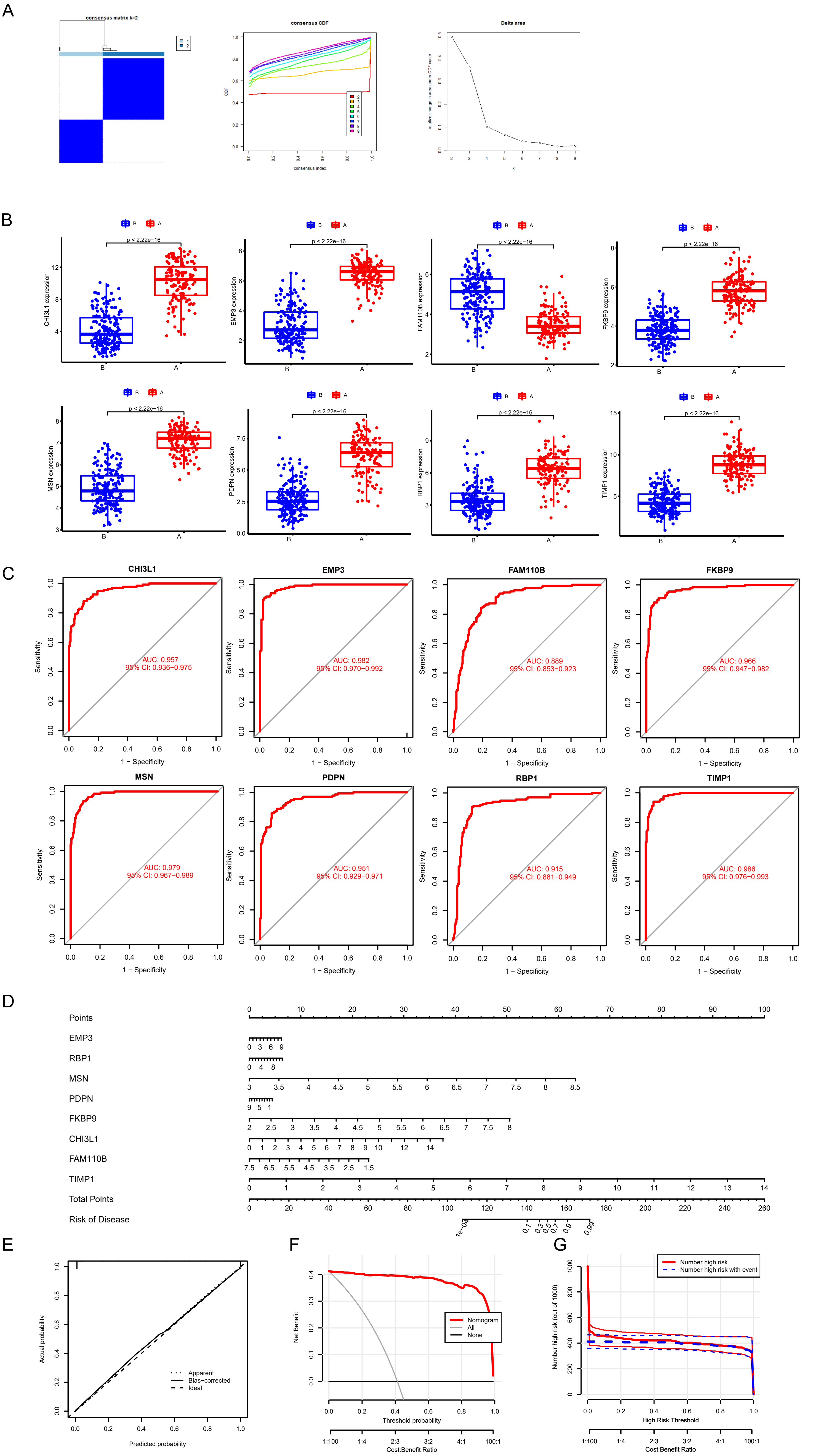

Supplementary figure 11. Validation of aging CAF related genes in external data sets (dataset ID: mRNAseq_325). (A) Consensus clustering for glioma samples based on the expression profiles of aging CAF related genes in the validation cohort (dataset ID: mRNAseq_325). (B) Comparisons of the expression levels of the eight featured genes between two gene clusters. (C) The ROC curves demonstrated the high accuracy of the eight featured genes for discriminating the two gene clusters. (D) A nomogram model was built to predict the probability of gene cluster A based on the expression of these featured genes. (E) The calibration curve revealed the accuracy of the nomogram model. (F) DCA of the nomogram model. (G) Clinical impact curves of the nomogram model.
