# Supplementary material for: Characterization of aging cancer-associated fibroblasts draws implications in prognosis and immunotherapy response in low-grade gliomas
Source: Front Genet. 2022 Aug 24;13:897083. doi: 10.3389/fgene.2022.897083 (PMC9449154; doi:10.3389/fgene.2022.897083)
Supplement: Supplementary file 11 [file DataSheet3.PDF]

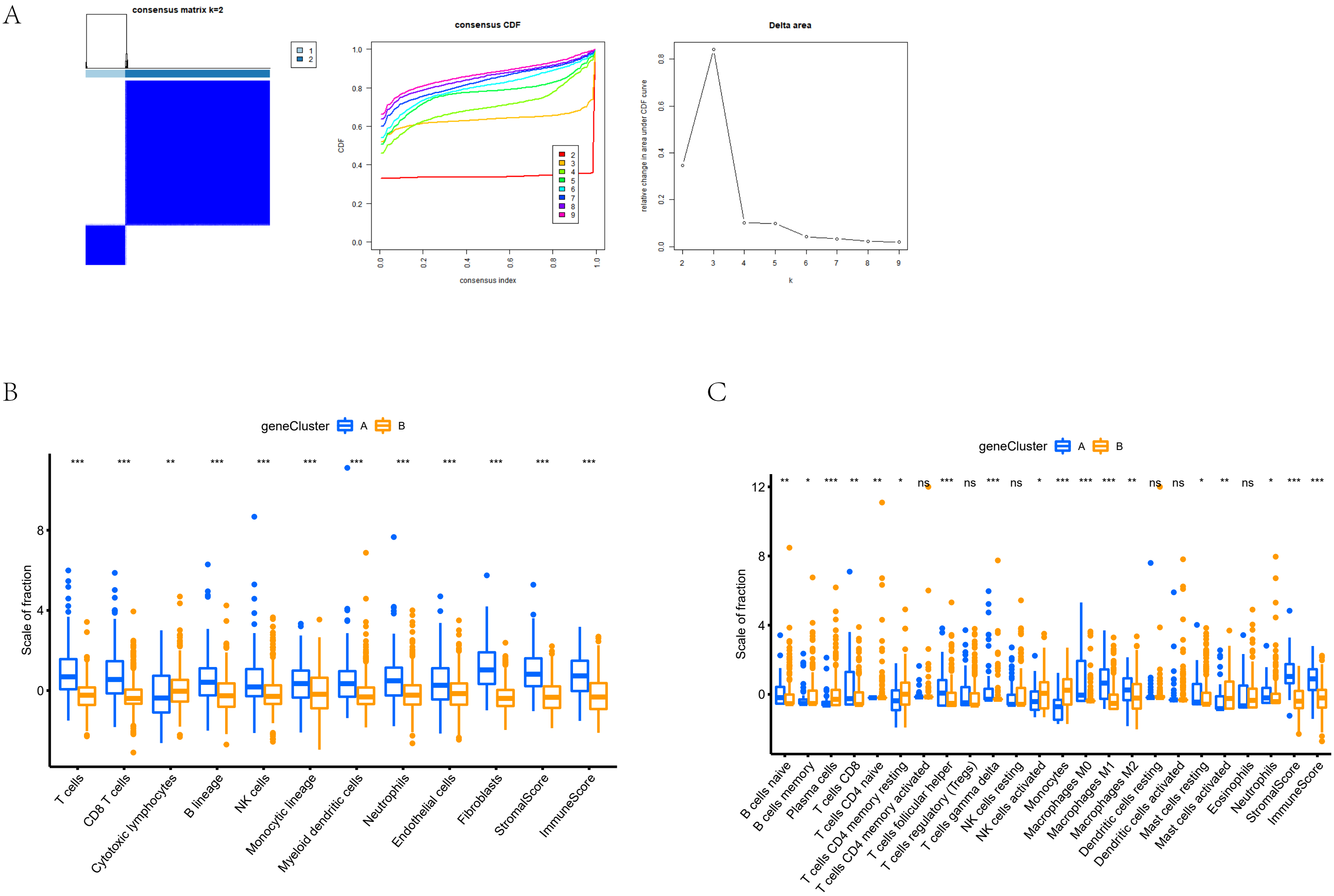

Supplementary figure 3. (A) Classification of LGG samples based on the expression profiles of ACAFRGs via consensus clustering method. (B) Comparisons of TME components between two gene clusters which were calculated by MCP counter method. (C) Comparisons of the immune cells between two gene clusters which were calculated by CIBERSORT algorithm. TME, tumor microenvironment. * means $p < 0.05$, ** means $p < 0.01$ and ***means $p < 0.001$.
