# Supplementary material for: Characterization of aging cancer-associated fibroblasts draws implications in prognosis and immunotherapy response in low-grade gliomas
Source: Front Genet. 2022 Aug 24;13:897083. doi: 10.3389/fgene.2022.897083 (PMC9449154; doi:10.3389/fgene.2022.897083)
Supplement: Supplementary file 12 [file Table2.DOCX]

**Supplementary table 2. Clinicopathological features of LGG patients in CGGA database (DataSet ID: mRNA-array_301)**

| Features |  | Total | High-aging  CAFscore | Low-aging  CAFscore |
| --- | --- | --- | --- | --- |
| TCGA  subtypes | Classical | 7(4.4%) | 4(16.67%) | 3(2.22%) |
|  | Mesenchymal | 31(19.5%) | 15(62.5%) | 16(11.85%) |
|  | Neural | 62(38.99%) | 5(20.83%) | 57(42.22%) |
|  | Proneural | 59(37.11%) | 0(0%) | 59(43.7%) |
| Type | Primary | 143(89.94%) | 19(79.17%) | 124(91.85%) |
|  | Recurrent | 16(10.06%) | 5(20.83%) | 11(8.15%) |
| Grade | G2 | 106(66.67%) | 9(37.5%) | 97(71.85%) |
|  | G3 | 53(33.33%) | 15(62.5%) | 38(28.15%) |
| Gender | Female | 69(43.4%) | 8(33.33%) | 61(45.19%) |
|  | Male | 90(56.6%) | 16(66.67%) | 74(54.81%) |
| Age | <45 | 111(70.7%) | 14(60.87%) | 97(72.39%) |
|  | >=45 | 46(29.3%) | 9(39.13%) | 37(27.61%) |
| Radio  status | treated | 136(86.62%) | 18(75%) | 118(88.72%) |
|  | untreated | 21(13.38%) | 6(25%) | 15(11.28%) |
| Chemo  status | treated | 69(44.81%) | 18(75%) | 51(39.23%) |
|  | untreated | 85(55.19%) | 6(25%) | 79(60.77%) |
| IDH  mutation | Mutant | 105(66.46%) | 7(29.17%) | 98(73.13%) |
|  | Wildtype | 53(33.54%) | 17(70.83%) | 36(26.87%) |
| 1p19q  Codeletion | Codel | 16(32%) | 0(0%) | 16(38.1%) |
|  | Non-codel | 34(68%) | 8(100%) | 26(61.9%) |
| MGMTp  methylation | methylated | 43(28.67%) | 5(23.81%) | 38(29.46%) |
|  | un-methylated | 107(71.33%) | 16(76.19%) | 91(70.54%) |
