# Supplementary material for: Characterization of aging cancer-associated fibroblasts draws implications in prognosis and immunotherapy response in low-grade gliomas
Source: Front Genet. 2022 Aug 24;13:897083. doi: 10.3389/fgene.2022.897083 (PMC9449154; doi:10.3389/fgene.2022.897083)
Supplement: Supplementary file 13 [file DataSheet1.PDF]

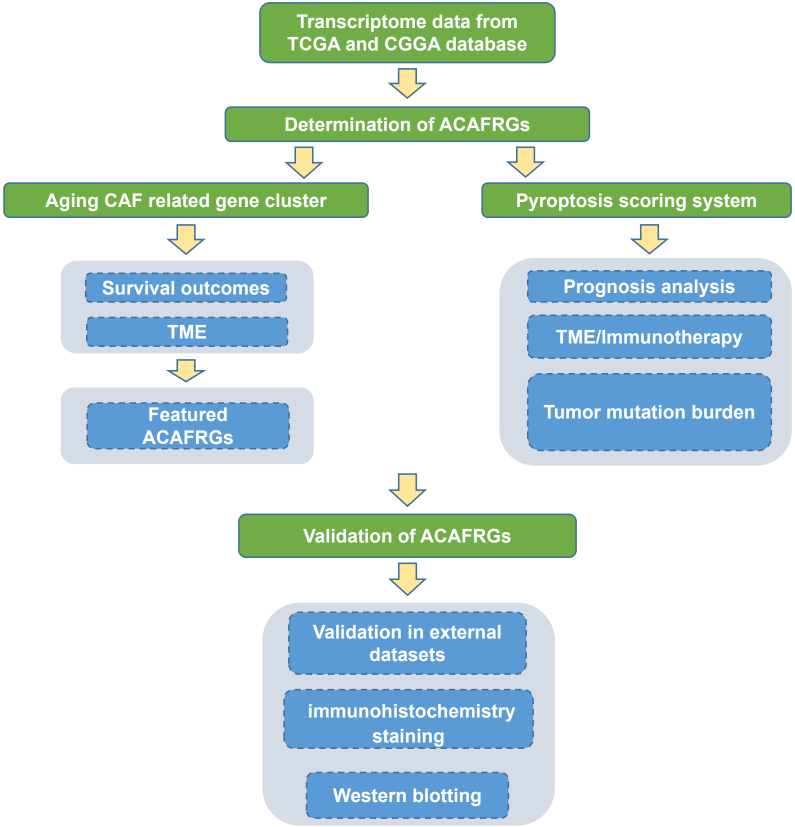

Supplementary figure 1. Schematic diagram of the workflow of this study. ACAFRGs, aging cancer associated fibroblast related genes; CAF, cancer associated fibroblast; TME, tumor microenvironment.
