# Supplementary material for: Characterization of aging cancer-associated fibroblasts draws implications in prognosis and immunotherapy response in low-grade gliomas
Source: Front Genet. 2022 Aug 24;13:897083. doi: 10.3389/fgene.2022.897083 (PMC9449154; doi:10.3389/fgene.2022.897083)
Supplement: Supplementary file 14 [file DataSheet5.PDF]

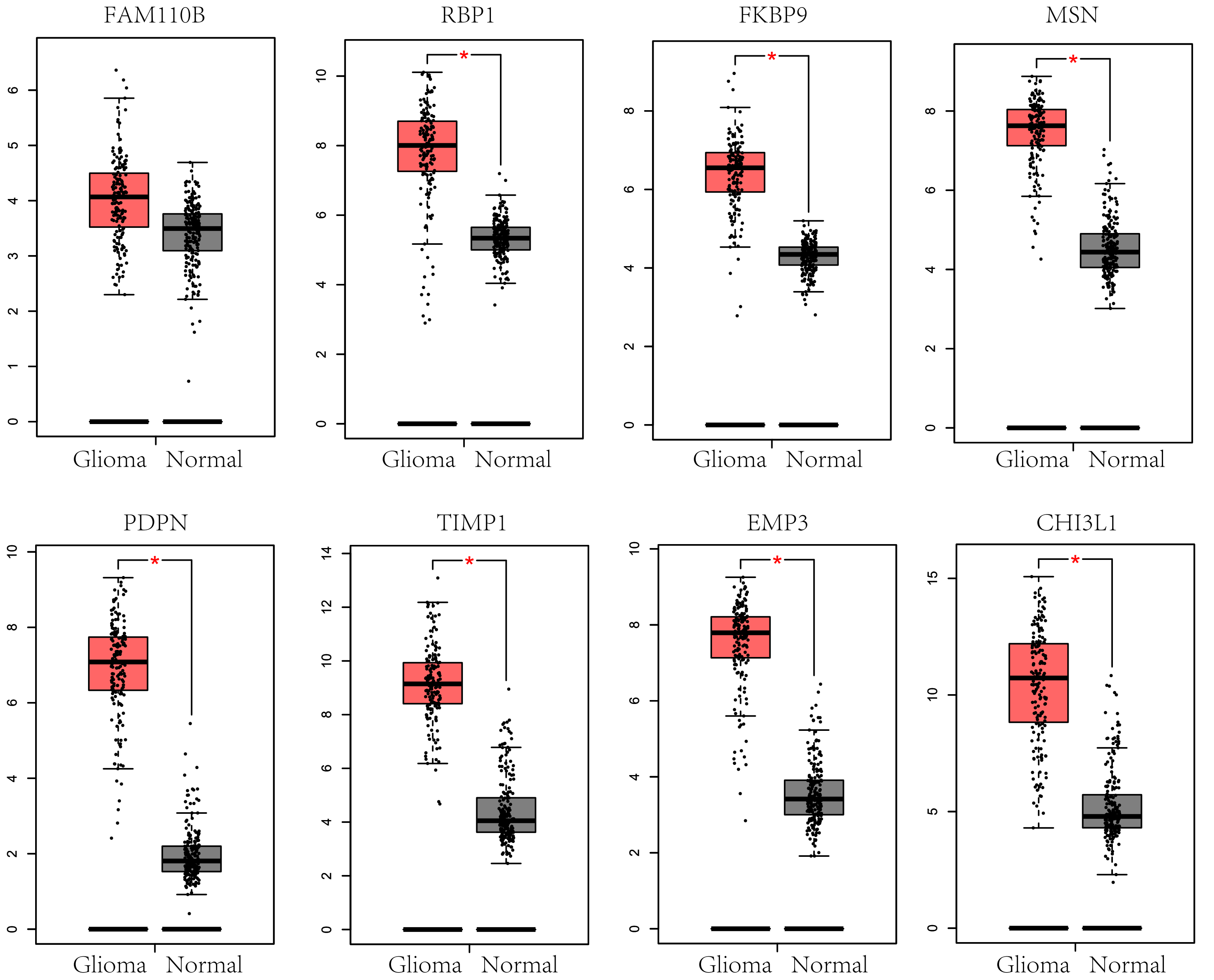

Supplementary figure 5. The differential expression patterns of the 8 featured genes between gliomas and normal samples were analyzed through the GEPIA online tools(GEPIA, Gene Expression Profiling Interactive Analysis, http://gepia.cancer-pku.cn/).
