# Supplementary material for: Characterization of aging cancer-associated fibroblasts draws implications in prognosis and immunotherapy response in low-grade gliomas
Source: Front Genet. 2022 Aug 24;13:897083. doi: 10.3389/fgene.2022.897083 (PMC9449154; doi:10.3389/fgene.2022.897083)
Supplement: Supplementary file 15 [file DataSheet12.PDF]

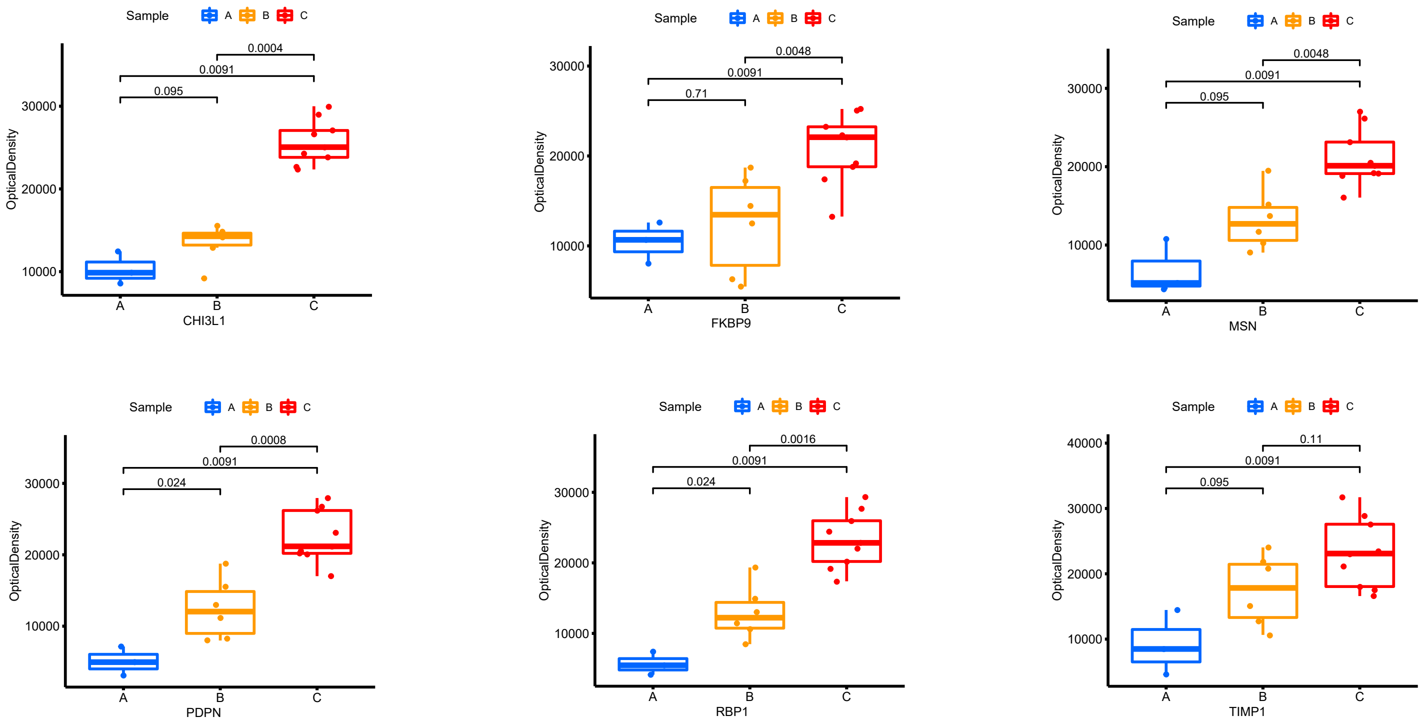

Supplementary figure 12. Comparisons of the optical density of the bands in the western blotting between different samles. A represented normal brain samples; B represented G2 glioma samples; C represented G3 glioma samples.
