# Supplementary material for: Characterization of aging cancer-associated fibroblasts draws implications in prognosis and immunotherapy response in low-grade gliomas
Source: Front Genet. 2022 Aug 24;13:897083. doi: 10.3389/fgene.2022.897083 (PMC9449154; doi:10.3389/fgene.2022.897083)
Supplement: Supplementary file 16 [file Table4.DOCX]

**Supplementary table 4. Calculation for risk score from multivariate cox regression analysis based on ACAFRGs**

| **Gene** | **coef** | **HR** | **HR.95L** | **HR.95H** | **P value** |
| --- | --- | --- | --- | --- | --- |
| **IGFBP2** | 0.17604571 | 1.192492566 | 0.983582609 | 1.445774364 | 0.073211847 |
| **EMP3** | 0.3273485 | 1.387284862 | 1.088315332 | 1.768383879 | 0.008208634 |
| **EN1** | 0.193577847 | 1.213583857 | 1.04347574 | 1.411423113 | 0.01199551 |
| **SH2D4A** | 0.349144992 | 1.417854753 | 1.059822428 | 1.896838608 | 0.01871127 |
| **GJC1** | 0.432912257 | 1.541740937 | 1.204429205 | 1.973519994 | 0.000589298 |
| **TNFRSF11B** | 0.36343699 | 1.438264229 | 1.139668352 | 1.815092951 | 0.002205052 |
| **CHI3L1** | -0.120776221 | 0.886232257 | 0.789612147 | 0.994675192 | 0.040304607 |
| **VAV3** | -0.373300065 | 0.688458619 | 0.536247165 | 0.883874641 | 0.003408668 |
| **KIF2C** | 0.35636642 | 1.428130749 | 1.16916397 | 1.744457996 | 0.000481263 |
| **TNFAIP6** | 0.171782248 | 1.187419242 | 0.980444969 | 1.438086279 | 0.07876927 |
| **GPX8** | -0.281262725 | 0.754829996 | 0.561221555 | 1.015228868 | 0.062883632 |
| **PPIC** | 0.624989416 | 1.868226183 | 1.411191687 | 2.473277801 | 1.26E-05 |
| **BMP2** | -0.273206438 | 0.760935685 | 0.620475318 | 0.933192827 | 0.008688699 |
| **GALNT13** | 0.479526278 | 1.615309014 | 1.297571355 | 2.010851426 | 1.78E-05 |
| **TMEM71** | 0.386707478 | 1.472125798 | 1.119388619 | 1.936016079 | 0.005658568 |
| **CHRNA1** | 0.223892634 | 1.250936704 | 1.075750444 | 1.454652097 | 0.003631356 |
| **OCIAD2** | 0.218968403 | 1.244791945 | 1.004206042 | 1.543016991 | 0.045687712 |
| **EMILIN2** | -0.366920357 | 0.692864825 | 0.549009808 | 0.87441364 | 0.002000113 |
| **RBP1** | -0.212548197 | 0.808521347 | 0.666738763 | 0.980454121 | 0.030724576 |
| **ALDOC** | -0.348938488 | 0.705436522 | 0.570505524 | 0.872280223 | 0.001275174 |
| **SLC25A24** | 0.239667695 | 1.270826779 | 0.911661096 | 1.771492398 | 0.157297062 |
| **H19** | 0.222062393 | 1.248649283 | 1.117957225 | 1.394619577 | 8.26E-05 |
| **BCAT1** | -0.178555809 | 0.836477373 | 0.669874753 | 1.04451525 | 0.115109223 |
| **FAM114A1** | -0.313935286 | 0.730566304 | 0.515213455 | 1.035933981 | 0.078096751 |
| **TOX** | -0.285295132 | 0.751792344 | 0.615713117 | 0.917946544 | 0.005104955 |
| **TRIP6** | 0.642368579 | 1.900978167 | 1.388417432 | 2.602760459 | 6.15E-05 |
| **SERPINH1** | -0.343259297 | 0.709454229 | 0.513526979 | 0.980134098 | 0.037374369 |
| **F11R** | -0.303248029 | 0.738415925 | 0.529463231 | 1.029831812 | 0.07397577 |
| **EMILIN3** | -0.114911607 | 0.891444938 | 0.784052106 | 1.013547532 | 0.079344044 |
| **AQP1** | 0.088314348 | 1.09233144 | 0.978272595 | 1.219688644 | 0.116517778 |
| **KHNYN** | 0.300392012 | 1.350388072 | 0.968112507 | 1.883611596 | 0.076875763 |
| **MARVELD1** | -0.461338558 | 0.630439201 | 0.499578083 | 0.795578508 | 0.000101697 |
| **TXLNB** | -0.172481561 | 0.841573806 | 0.698980345 | 1.013256632 | 0.068617496 |
| **FAM183A** | -0.128540935 | 0.879377564 | 0.75467906 | 1.024680478 | 0.099459364 |
| **NPNT** | -0.131142699 | 0.877092605 | 0.748243202 | 1.028130207 | 0.105717115 |
| **SEL1L3** | 0.323732705 | 1.382277782 | 1.185715013 | 1.611425887 | 3.52E-05 |
| **SOCS1** | -0.203825939 | 0.815604324 | 0.623678309 | 1.066592189 | 0.136486595 |
| **LGALS1** | -0.27173363 | 0.762057224 | 0.562402476 | 1.03259007 | 0.079590776 |
| **COL5A2** | -0.457499404 | 0.632864206 | 0.505345371 | 0.792561141 | 6.75E-05 |
| **MT1M** | 0.236875569 | 1.267283419 | 1.048440367 | 1.531806017 | 0.014324072 |
| **PCDH15** | -0.169501642 | 0.844085369 | 0.709694944 | 1.003924455 | 0.055403694 |
| **TCEA3** | -0.248539792 | 0.779938825 | 0.602127334 | 1.010259019 | 0.05974789 |
