# Supplementary material for: Characterization of aging cancer-associated fibroblasts draws implications in prognosis and immunotherapy response in low-grade gliomas
Source: Front Genet. 2022 Aug 24;13:897083. doi: 10.3389/fgene.2022.897083 (PMC9449154; doi:10.3389/fgene.2022.897083)
Supplement: Supplementary file 17 [file DataSheet8.PDF]

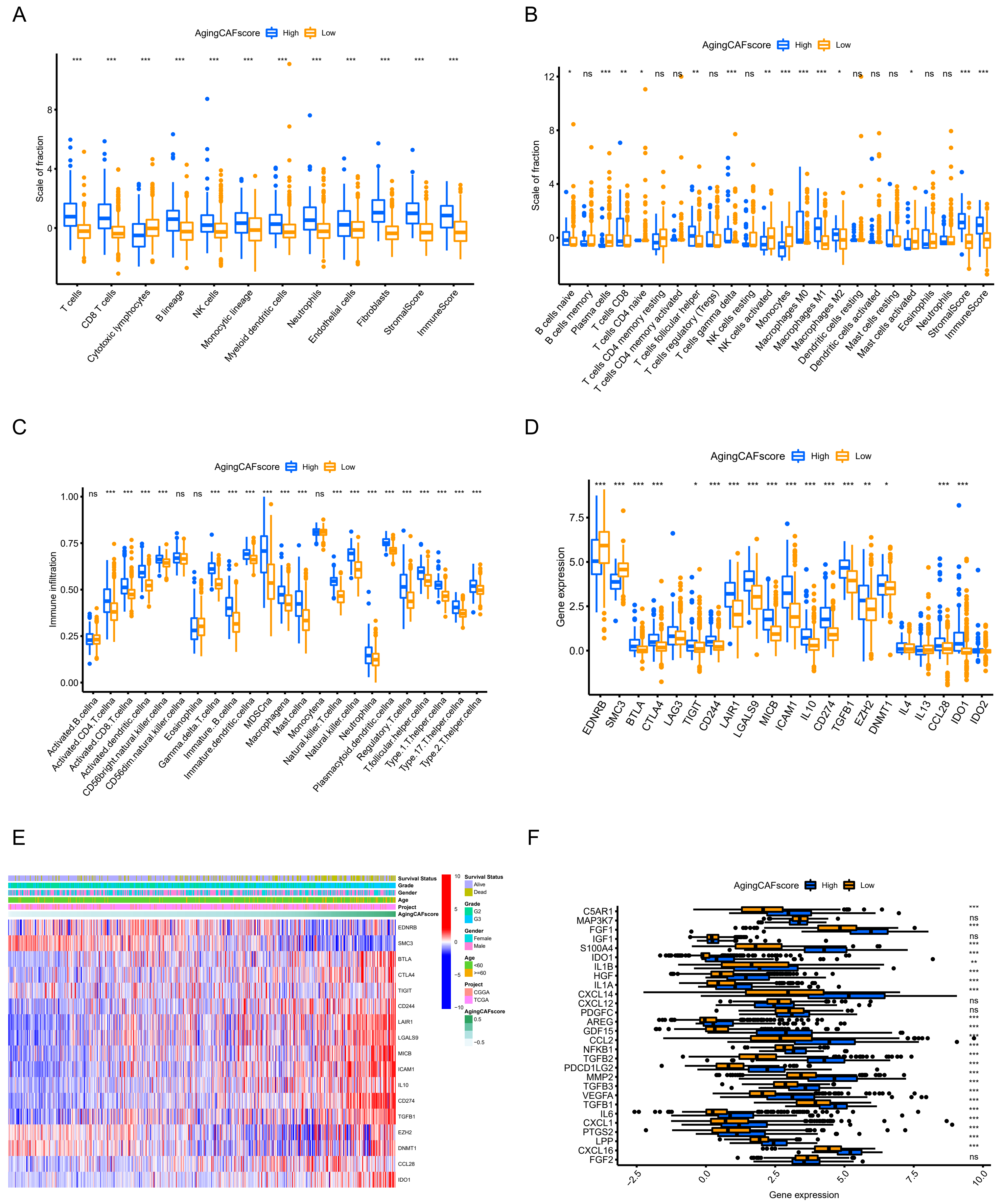

Supplementary figure 8. (A-C) Comparisons of TME components between two groups by using MCP counter algorithm (A), CIBERSORT algorithm (B) and ssGSEA method (C), respectively. (D) Comparisons of the expression levels of genes negatively regulating The Cancer-Immunity Cycle between two groups. (E) The expression patterns of genes negatively regulating The Cancer-Immunity Cycle with increasing of aging CAF score. (F) Comparisons of the expression levels of cytokines secreted by CAFs between two groups. * means $p < 0.05$, ** means $p < 0.01$ and ***means $p < 0.001$. TME, tumor microenvironment; CAF, cancer associated fibroblast.
